# Autosurv: interpretable deep learning framework for cancer survival analysis incorporating clinical and multi-omics data

**DOI:** 10.1038/s41698-023-00494-6

**Published:** 2024-01-05

**Authors:** Lindong Jiang, Chao Xu, Yuntong Bai, Anqi Liu, Yun Gong, Yu-Ping Wang, Hong-Wen Deng

**Affiliations:** 1 Tulane Center of Biomedical Informatics and Genomics, School of Medicine, Tulane University, New Orleans, LA 70112 USA; 2 Department of Biostatistics and Epidemiology, University of Oklahoma Health Sciences Center, Oklahoma City, OK 73104 USA; 3 Department of Biomedical Engineering, School of Science and Engineering, Tulane University, New Orleans, LA 70118 USA

**Keywords:** Computational biology and bioinformatics, Cancer

## Abstract

Accurate prognosis for cancer patients can provide critical information for optimizing treatment plans and improving life quality. Combining omics data and demographic/clinical information can offer a more comprehensive view of cancer prognosis than using omics or clinical data alone and can also reveal the underlying disease mechanisms at the molecular level. In this study, we developed and validated a deep learning framework to extract information from high-dimensional gene expression and miRNA expression data and conduct prognosis prediction for breast cancer and ovarian-cancer patients using multiple independent multi-omics datasets. Our model achieved significantly better prognosis prediction than the current machine learning and deep learning approaches in various settings. Moreover, an interpretation method was applied to tackle the “black-box” nature of deep neural networks and we identified features (i.e., genes, miRNA, demographic/clinical variables) that were important to distinguish predicted high- and low-risk patients. The significance of the identified features was partially supported by previous studies.

## Introduction

Cancer is one of the leading causes of death worldwide^1^. It is estimated that in 2022, 1,918,030 new cases will be diagnosed, and about 609,360 people will die from cancer (i.e., almost 1700 deaths per day) in the United States^2^. Accurate cancer prognosis prediction helps clinicians to conduct more appropriate treatment allocation for patients to prolong life span, increase life quality, and reduce unnecessary treatment cost. Recent studies have applied machine learning (ML) techniques in the analysis of clinical and genomic features, and they showed that ML has improved performance in cancer susceptibility, recurrence, and survival prediction compared to traditional approaches (e.g., Kaplan–Meier method)^3–5^. In practice, several issues can undermine the robustness of survival predictions. Firstly, measuring some important clinical variables (e.g., disease stage) relies heavily on the clinician’s individual interpretation, which may introduce human bias, thereby reducing the accuracy and reliability of the prediction results^1^. Interestingly, the study^5^ showed that ML model can give more accurate predictions than the attending physicians in cancer survival analysis. Secondly, small sample size accompanied by high-dimensional input data (e.g., gene expression data, whole slide image, etc.) can result in overfitting^6^ and hamper the generalizability of existing models. Thirdly, the relationship between predictors and survival outcome may be non-linear^7^, and thus existing models that assume a linear relationship (e.g., Cox Proportional Hazards model^8^) may produce inaccurate results.

Following the widespread application of high-throughput sequencing technologies, omics data (e.g., mRNA expression data, miRNA expression data) have become more accessible than ever. Incorporating omics information in analyses could provide models with a more comprehensive view and mitigate the bias that may be brought by a single data type. Furthermore, this could help us understand disease mechanisms at the molecular level. Some recent studies included omics information in their models for cancer classification or prognosis^1,6,9–15^; moreover, several studies^11,13–15^ have shown that integrating multi-omics data can improve model performance compared with single omics. Thus, efficient, and effective incorporation of multi-omics data into cancer survival analysis is worth further investigation.

There are many approaches available for survival analysis, such as the Cox proportional hazard (CoxPH) and related models^8,16,17^, random survival forest (RSF)^18^, Extreme Gradient Boosting (XGBoost) with Accelerated Failure Time (AFT) (XGB-AFT)^19^ and some newly developed Deep Neural Networks (DNN)^1,6,9–12,14,20^. CoxPH model assumes a linear relationship between a patient’s log-risk of failure and covariates^8,20^. Although it is commonly applied for survival analysis, the CoxPH model cannot handle complex data structure well^6^. In addition, due to the high-dimension-low-sample-size issue^6^ commonly seen in analyses with omics data, directly applying CoxPH on omics data can cause overfit. Regularization techniques like LASSO or elastic net can help lower the risk of overfitting by conducting variable selection. On the other hand, random survival forest is an ensemble tree method that extends upon Breiman’s random forest (RF) method^21^ for the analysis of right-censored survival data. It handles nonlinearity automatically, can produce highly accurate ensemble predictors, and can offer nearly unbiased error rate estimates even in the presence of significant amounts of missing data. Gradient boosting is another ensemble-based method, and XGBoost is a library for efficient implementation of its algorithm^22^. Generally, random forest can lower the chance of overfitting, while gradient boosting can reduce the risk of underfitting, and XGBoost has helped many winner teams in Kaggle structured data competitions^22^. XGB-AFT^19^ adapted the AFT model to integrate with XGBoost, it captures non-linear data patterns like other ML approaches and produces survival time estimates directly. Moreover, both RSF and XGB-AFT are not restricted by the proportional hazard assumption.

As a trending approach, DNN can also deal with non-linear relationships intrinsically, which can well represent complicated data structures. Owing to their flexibility, deep learning (DL) models can be designed (or combined with other approaches) to conduct feature extraction and integration from high-dimensional omics data. DeepSURV^20^ is a model that is based on CoxPH but adopts a DNN structure. Katzman et al. demonstrated that DeepSURV was able to outperform the CoxPH model in prognosis prediction under various scenarios, highlighting the strength of deep learning models in handling complex data patterns compared to conventional approaches. In a similar fashion, Cox-nnet^1^ and CoxPASNet^6^ both adopted feed-forward DNN structures for prognosis prediction, but unlike DeepSURV, they can handle high-dimensional gene expression data. Moreover, CoxPASNet applies a sparse coding technique to further reduce the risk of overfitting. In the study^6^ CoxPASNet showed significantly better performance than Cox-nnet. OmiVAE is a DNN that combines gene expression and DNA methylation data for cancer classification^15^. OmiVAE consists of a variational autoencoder (VAE) and a downstream classification network. It can achieve task-oriented feature extraction and patient classification simultaneously in the supervised phase of its training scheme^15^. Zhang et al. showed that OmiVAE performed better when trained from multi-omics data than single-omics data. SALMON^11^ implemented local maximal Quasi-Clique Merger (lmQCM)^23^ for co-expression network analysis. The first principal components of the identified gene/miRNA co-expression modules were then extracted and input into a CoxPH Regression Network^11^. In their study, Huang et al. observed improved performance of SALMON when more omics data were incorporated. Cheerla et al. built a DL model that integrates multimodal representations from clinical, omics, and whole slide image data and performs pan-cancer prognosis prediction^10^. MultiSurv, proposed by ref. ^12^, is another DL-based pan-cancer prognosis prediction model. It also takes those three modalities as the input. But it applies a different integration approach (i.e., computing the row-wise maximum of the feature representation matrix) and does not depend on the proportional hazard assumption of the CoxPH model (i.e., owing to their implementation of the discrete-time survival model formulation). Finally, for result interpretation and feature importance investigation, previous DL approaches have applied gradient-based or perturbation-based methods^6,11,13^.

In this paper, we develop a deep learning model for prognosis prediction, namely, AUTOSurv. This model uses multi-omics data and tackles the high-dimension-low-sample-size issue through dimension reduction leveraging a specially designed VAE. We demonstrate that by virtue of its network structure and learning strategy, AUTOSurv obtained significantly better prognosis prediction performance compared to other existing modeling strategies and/or machine learning methods in various cases using multiple independent datasets. Furthermore, the strengths and weakness of different feature extraction, dimension reduction, and data integration approaches are deliberated. To resolve the “black-box” nature of DNNs, we applied the DeepSHAP interpretation approach^24–26^ to the learned AUTOSurv model and identified important genes, miRNA and pathways that contributed to distinguishing between high- and low-risk patients. We hope our work could be a step towards the development of more advanced deep learning approaches that not only can provide accurate prognosis prediction but also can unravel hidden mechanisms underlying cancer progression.

## Results

### AUTOSurv on multi-omics data integration

**Fig. 1:**
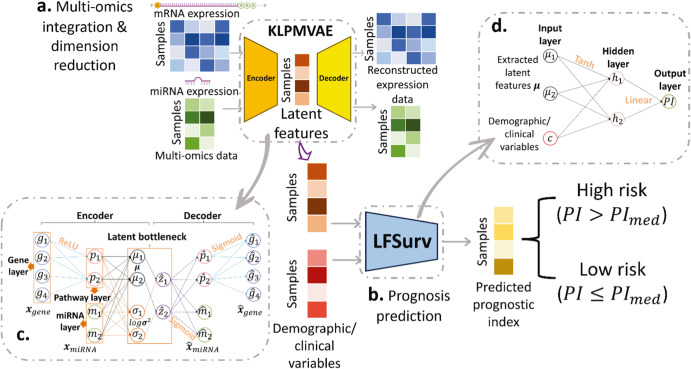
AUTOSurv workflow and key components illustration. **a** KL-PMVAE was trained to conduct integration and dimension reduction on gene expression and miRNA expression data. **b** Latent features generated by KL-PMVAE will be combined with the demographic/clinical variables and fed into the LFSurv network. The output of LFSurv will be a prognostic index (${PI}$) for each patient that reflects the patient’s risk of death. ${{PI}}_{{med}}$: median prognostic index. **c** Illustration of KL-PMVAE. The VAE model consists of an encoder and a decoder. The encoder has one gene layer (each node represents a gene), one pathway layer (each node represents a pathway), and one miRNA layer (each node represents a miRNA) and learns a distribution estimate of the latent variables $z$ (parameterized by means $\mu$ and variances ${\sigma }^{2}$ which were stored in the latent bottleneck). The decoder takes a sample $\hat{z}$ from the distribution estimate as input and outputs the reconstructed expression data ${\hat{x}}_{{miRNA}}$ and ${\hat{x}}_{{gene}}$. **d** Illustration of LFSurv. This network consists of an input layer, a hidden layer, and an output layer with only one node. The extracted latent features $\mu$ were concatenated with the demographic/clinical variables. The network receives the concatenated features and outputs the prognostic index (${PI}$).

The structure of AUTOSurv was presented in Fig. 1 and the “Methods” section. In general, the AUTOSurv model conducts prognosis prediction in two steps: (1) A pathway-information-guided VAE model with KL-annealing learning strategy (KL-PMVAE) extracts low-dimensional latent features from high-dimensional gene expression and miRNA expression data jointly; (2) A multi-layer perceptron network (LFSurv) takes the concatenation of the latent features from KL-PMVAE and demographic/clinical variables as input and computes prognostic index (*PI*) for each patient, where higher *PI* implies higher risk of death. During the developmental stage, we examined different structures and learning strategies of AUTOSurv and compared them with other deep learning approaches to finalize the proposed model. In this stage, we used The Cancer Genome Atlas (TCGA) Breast (BRCA) and Ovarian (OV) cancer multi-omics datasets (data collection and preprocessing details listed in Methods), and three different cases were designed for performance evaluation. In the first case (denoted as “mRNA + miRNA + clinical”), gene expression data, miRNA expression data, and demographic/clinical data (e.g., age, disease stage, race) were used as model input, and two strategies for multi-omics integration (“entangle” and “concatenate”) were analyzed. In the “entangle” strategy, which is the final strategy chosen for AUTOSurv, the KL-PMVAE section of AUTOSurv combines gene expression and miRNA expression information to derive a joint set of latent features (${{\boldsymbol{\mu }}}_{{gene}+{miRNA}}$) as input for LFSurv (see Fig. 1c for illustration). In the “concatenate” strategy, altered KL-PMVAE extracts latent features, ${{\boldsymbol{\mu }}}_{{gene}}$ and ${{\boldsymbol{\mu }}}_{{miRNA}}$, from gene and miRNA expression data respectively (Supplementary Fig. 2). LFSurv takes the direct concatenation of ${{\boldsymbol{\mu }}}_{{gene}}$ and ${{\boldsymbol{\mu }}}_{{miRNA}}$, instead of ${{\boldsymbol{\mu }}}_{{gene}+{miRNA}}$ as input. In the other two cases (denoted as “mRNA + clinical” and “miRNA + clinical”, respectively), demographic/clinical data, and a single type of omics data were used as model input. Gene expression data was used for “mRNA + clinical”; while miRNA expression data was used for “miRNA + clinical”. LFSurv takes either ${{\boldsymbol{\mu }}}_{{gene}}$ or ${{\boldsymbol{\mu }}}_{{miRNA}}$ (plus demographic/clinical data), as illustrated in Supplementary Fig. 2c. By comparing to “mRNA + miRNA + clinical”, we can examine the performance gain from multi-omics vs single-omics input features. Unless otherwise mentioned, KL-annealing was applied for all VAE-related model structures.

**Fig. 2:**
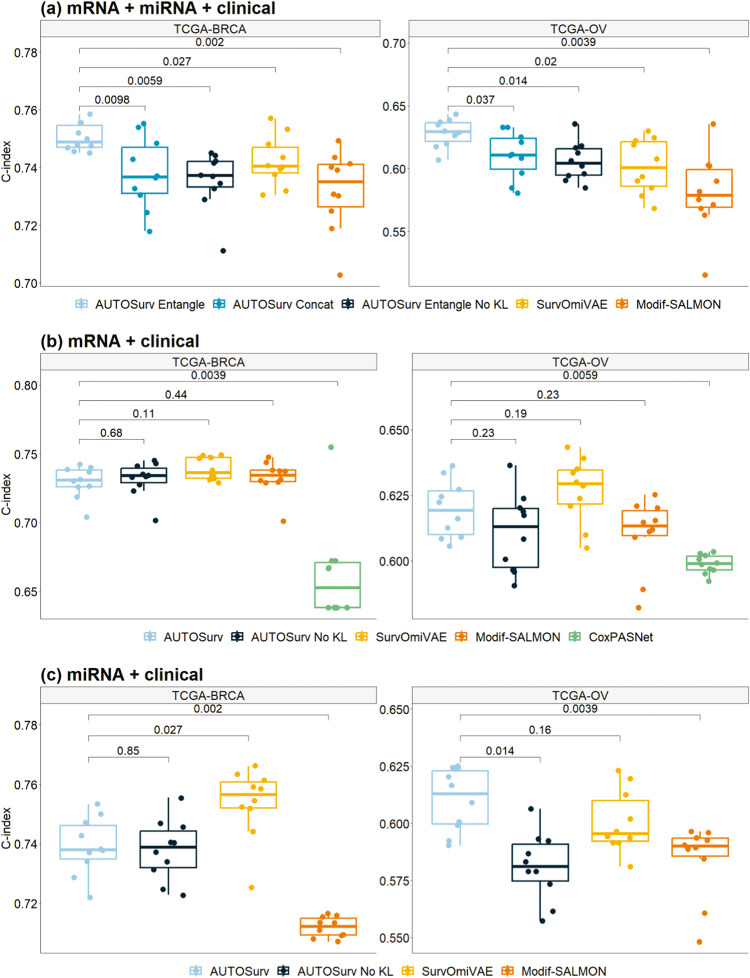
Boxplots for performance comparison between different models/model structures in terms of testing set C-index. Predictions measured on TCGA-BRCA and TCGA-OV datasets in three different cases: **a** mRNA + miRNA + clinical; **b** mRNA + clinical; **c** miRNA + clinical. AUTOSurv Entangle: AUTOSurv with “entangle” integration strategy; AUTOSurv Concat: AUTOSurv with “concatenate” integration strategy, more details about the alterations of AUTOSurv were illustrated in Supplementary Fig. 2; AUTOSurv Entangle No KL: AUTOSurv (with “entangle” integration strategy) without KL-annealing; Modif-SALMON: modified-SALMON. The *p*-value from two-sided Wilcoxon signed-rank test (null hypothesis ${{\rm{H}}}_{0}$: median difference is 0; versus alternative hypothesis ${{\rm{H}}}_{{\rm{A}}}$: median difference is not 0) is displayed between boxes.

As shown in Fig. 2, AUTOSurv with “entangle” integration strategy achieved best prediction performance for both TCGA-BRCA (median C-index = 0.749) and TCGA-OV (median C-index = 0.629) datasets. In the “mRNA + miRNA + clinical” case (Fig. 2a), compared to the “concatenate” strategy, the “entangle” strategy had superior performance in integrating two types of omics data for prognosis prediction in terms of C-index, with the significance of performance difference checked via two-sided Wilcoxon signed-rank test (for TCGA-BRCA: median C-index 0.749 vs 0.737, *p*-value = 0.010; for TCGA-OV: median C-index 0.629 vs 0.611, *p*-value = 0.037). When we compared the effectiveness of multi-omics data integration in “mRNA +miRNA + clinical” to the individual omics analysis in “mRNA + clinical” and “miRNA + clinical”, the “concatenate” strategy did not render better prediction performance (two-sided Wilcoxon signed-rank test returned *p*-value > 0.1 for all comparisons). One possible explanation is that, given miRNA mostly affects the phenotype by regulating the expression of certain genes, the survival-related-information underlying gene expression and miRNA expression data, respectively, may have some degree of overlap. Therefore, if concatenated directly, the presumably overlapped information in ${{\boldsymbol{\mu }}}_{{gene}}$ and ${{\boldsymbol{\mu }}}_{{miRNA}}$ can be redundant for LFSurv to predict prognosis. On the other hand, when the “entangle” strategy was applied, the decoder of KL-PMVAE was trained to reconstruct the two types of omics data simultaneously from a common set of latent features (see the “Methods” section). The aforementioned information overlap might facilitate utilization of crosstalk (especially the non-linear interactions) between the two omics data and help our model extract the most relevant information for the reconstruction task. Thus KL-PMVAE using “entangle” strategy in “mRNA + miRNA + clinical” achieved more efficient feature extraction than single-omics VAE models in terms of C-index (TCGA-BRCA “mRNA + miRNA + clinical” vs “mRNA + clinical”: median C-index 0.749 vs 0.731, *p*-value = 0.002; TCGA-BRCA “mRNA + miRNA + clinical” vs “miRNA + clinical”: median C-index 0.749 vs 0.738, *p*-value = 0.037; TCGA-OV “mRNA + miRNA + clinical” vs “mRNA + clinical”: median C-index 0.629 vs 0.619, *p*-value = 0.020; TCGA-OV “mRNA + miRNA + clinical” vs “miRNA + clinical”: median C-index 0.629 vs 0.613, *p*-value = 0.014; *p*-values obtained via two-sided Wilcoxon signed-rank tests).

We tested the KL-annealing learning strategy (see the “Methods” section) in AUTOSurv. When only single-omics data were used (Fig. 2b, c), the prediction performance of AUTOSurv with or without KL-annealing did not differ significantly in most scenarios. The exception is for the “miRNA + clinical” case of TCGA-OV dataset, where AUTOSurv with KL-annealing achieved significantly better performance (median C-index 0.613 vs 0.581, *p*-value = 0.014). When two omics data types were modeled simultaneously using “entangle” integration strategy in the “mRNA + miRNA + clinical” case, the performance of AUTOSurv with KL-annealing was significantly better (median C-index 0.749 vs 0.737, *p*-value = 0.006 for TCGA-BRCA; median C-index 0.629 vs 0.604, *p*-value = 0.014 for TCGA-OV). This implies that KL-annealing helped retain useful information in the latent features when the reconstruction task of KL-PMVAE became more complicated. Moreover, for AUTOSurv without KL-annealing, “mRNA + miRNA + clinical” performance (with “entangle” strategy) did not improve compared to “mRNA + clinical” or “miRNA + clinical” (except for “mRNA + miRNA + clinical” vs “miRNA + clinical” of TCGA-OV [median C-index 0.604 vs 0.581], *p*-value equals 0.009). Therefore, it is reasonable to assume that the combination of the “entangle” integration strategy and KL-annealing, instead of the “entangle” strategy alone, gave AUTOSurv better prediction performance when both omics data types were incorporated. These findings highlight the subtlety in selecting a plausible structure and optimization strategy when constructing deep neural networks. Moreover, the results encourage us to explore in future studies whether KL-annealing has the potential to boost the performance of VAE models in more complex integration tasks involving more than two types of omics data.

To assess the influence of omics data on prognosis prediction, we fitted LFSurv using only demographic/clinical variables and obtained testing set median C-index 0.714 for TCGA-BRCA dataset and 0.623 for TCGA-OV dataset, which are lower than those from AUTOSurv in the case “mRNA + miRNA + clinical” (median C-index 0.749 for TCGA-BRCA, *p*-value = 0.002; median C-index 0.629 for TCGA-OV dataset, *p*-value = 0.131). It suggests that incorporating omics information improved prediction performance of AUTOSurv, with greater advancement for the TCGA-BRCA dataset. This finding also implied that the amount of survival-related information embedded in omics data might vary across different cancer types, which may raise the issue of optimizing resource allocation to collect informative types of omics data and conduct cost-effective survival analysis/prediction for different cancer types. Nevertheless, considering the noticeable gap in sample size between the TCGA-BRCA and TCGA-OV datasets (i.e., 1058 vs 355), this statement may require further verification, which is out of the scope of this study.

We compared prognosis prediction performance of LFSurv network with the conventional multivariable CoxPH model using only demographic/clinical data (i.e., all clinical variables included as covariates in a single model). The linear combination of the covariates (denoted as log-risk function in ref. ^20^) in CoxPH model is equivalent to *PI* in AUTOSurv, and higher value of the log-risk function implies higher risk of death. For each dataset, we trained a CoxPH model using the whole tuning set and applied the trained model to the testing set (see the “Methods” section for more details about data division). The log-risk function estimates for the testing set patients combined with their overall follow-up times and event indicators were used to calculate testing set C-index following Eq. (5) in the “Methods” section, and we obtained testing set C-index of 0.673 (*p*-value = 0.002 vs LFSurv) and 0.606 (*p*-value = 0.041 vs LFSurv) for TCGA-BRCA dataset and TCGA-OV dataset respectively (because no hyperparameter was applied for the CoxPH model, in each dataset we only have to train the model once obtaining one testing set C-index). This implied that some higher-order interactions between the demographic/clinical variables captured by the hidden layer of LFSurv are potentially important for survival analysis. It showed the strength of deep neural networks in utilizing more complex data structure/feature relationships compared to the conventional CoxPH model.

### AUTOSurv compared to other deep learning approaches

We applied and adapted other recently developed and representative deep learning methods (CoxPASNet^6^, OmiVAE^15^, and SALMON^11^) under the same three cases as mentioned earlier and compared their performance with AUTOSurv. For the case “mRNA + miRNA + clinical”, we focus on the comparisons involving AUTOSurv [“entangle” strategy]. The results are summarized in Fig. 2.

The end-to-end DNN CoxPASNet was not originally designed to handle multi-omics data, so we used only gene expression data and demographic/clinical variables (the case “mRNA + clinical”) for this model. The testing set median C-index for CoxPASNet was 0.653 for the TCGA-BRCA dataset and 0.599 for the TCGA-OV dataset, which was significantly lower than that obtained by AUTOSurv (TCGA-BRCA: median C-index 0.731, *p*-value = 0.004; TCGA-OV: median C-index 0.619, *p*-value = 0.006) using the same input data. This implied that dropout combined with sparse coding might not be efficient enough when dealing with high-dimensional omics features in an end-to-end feed-forward deep neural network.

As mentioned in the Introduction, OmiVAE is another end-to-end deep learning model. Here we tailored OmiVAE to survival analysis as Surv-OmiVAE, which connects the encoder of KL-PMVAE to LFSurv and trains them together to achieve “task-oriented feature extraction” in its supervised phase^15^. From Fig. 2 we see that Surv-OmiVAE achieved high performance in single-omics cases (i.e., “mRNA + clinical” of TCGA-OV; “miRNA + clinical” of TCGA-BRCA). In the case “miRNA + clinical” of TCGA-BRCA dataset, Surv-OmiVAE outperformed AUTOSurv (median C-index 0.756 vs 0.738, *p*-value = 0.027). When multi-omics data were considered, however, AUTOSurv was able to beat Surv-OmiVAE (TCGA-BRCA: median C-index 0.749 vs 0.740, *p*-value = 0.027; TCGA-OV: median C-index 0.629 vs 0.601, *p*-value = 0.020). Furthermore, Surv-OmiVAE did not gain improvement in performance from multi-omics input features compared to single omics. Overall, our findings suggest that “task-oriented feature extraction” can potentially help capture survival-related information in the latent features and hence increase the prediction accuracy of LFSurv. Nevertheless, further adaptations are needed to accommodate multi-omics scenarios and make full use of information from different omics types.

For our implementation of SALMON, the widely applied WGCNA approach^27^ was adopted for co-expression network analysis. The first principal components of the identified gene/miRNA co-expression modules were taken as eigengenes/eigen-miRNAs and input into LFSurv for prognosis prediction. Figure 2 shows that AUTOSurv achieved comparable or better performance compared to the modified-SALMON when single omics data were used as input in the cases “mRNA + clinical” and “miRNA + clinical”. This implied that VAE could be more powerful than “WGCNA + PCA” in dimension reduction for certain types of expression data (i.e., miRNA expression data). In the case “mRNA + miRNA + clinical” for modified-SALMON, the eigengenes and eigen-miRNAs were concatenated and fed to LFSurv; not surprisingly, the performance did not improve compared to “mRNA + clinical” where only eigengenes were incorporated (median C-index 0.735 vs 0.734, *p*-value = 0.492 for TCGA-BRCA; median C-index 0.579 vs 0.613, *p*-value = 0.019 for TCGA-OV). This could be due to the same information overlap between the two omics data types mentioned above.

### Framework evaluation and benchmarking with machine learning methods on non-TCGA datasets

We assessed AUTOSurv framework on two non-TCGA independent datasets, the Caldas 2007 Breast Cancer (Caldas-BC) dataset and the ICGC – Ovarian Cancer Australian (ICGC-OVAU) dataset, and compared it with other machine learning survival analysis approaches (i.e., Cox Proportional Hazard model with Elastic Net [CoxPH-ENet], Random Survival Forest [RSF], Extreme Gradient Boosting with CoxPH [XGB-CoxPH]^28^, and Extreme Gradient Boosting with Accelerated Failure Time (XGB-AFT)). For AUTOSurv, KL-annealing was always applied, and “entangle” strategy was implemented for the “mRNA + miRNA + clinical” case in the ICGC-OVAU dataset. For the machine learning approaches, “WGCNA + PCA” procedure was implemented to reduce the dimension of gene/miRNA expression data, and we used the eigengenes/eigen-miRNAs for model fitting.

**Fig. 3:**
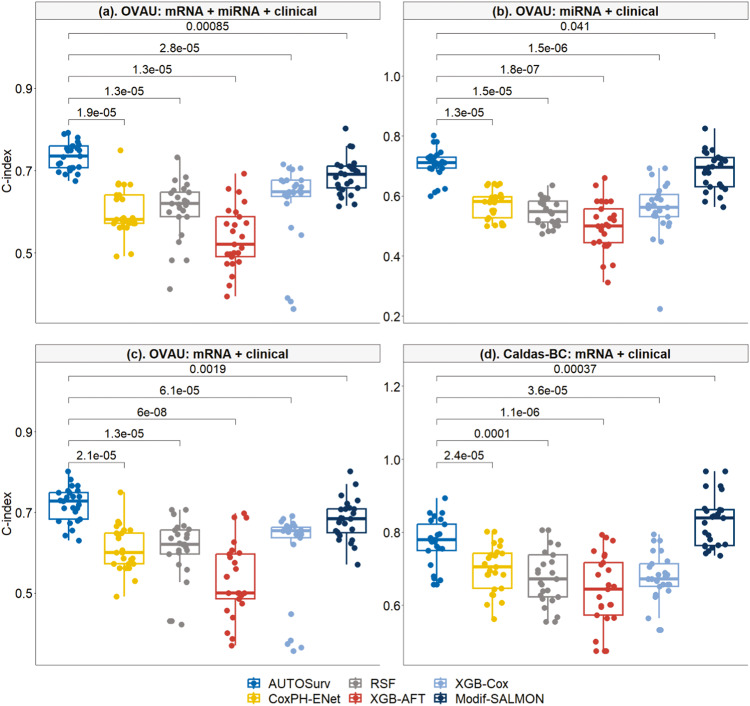
Performance comparison between AUTOSurv and other machine learning methods in two non-TCGA datasets: ICGC-OVAU and Caldas-BC. **a** the “mRNA + miRNA + clinical” case in ICGC-OVAU dataset; **b** the “miRNA + clinical” case in ICGC-OVAU dataset; **c** the “mRNA + clinical” case in ICGC-OVAU dataset; **d** the “mRNA + clinical” case in Caldas-BC dataset, which does not have miRNA expression data. CoxPH-ENet Cox Proportional Hazard model with Elastic Net, RSF Random Survival Forest, XGB-AFT Extreme Gradient Boosting with Accelerated Failure Time, XGB-Cox Extreme Gradient Boosting with CoxPH. The *p*-value from two-sided Wilcoxon signed-rank test (i.e., null hypothesis ${{\rm{H}}}_{0}$: median difference is equal to 0; versus alternative hypothesis ${{\rm{H}}}_{{\rm{A}}}$: median difference is not 0) is displayed between boxes.

From Fig. 3 we observe that AUTOSurv outperformed all the ML methods significantly for all three cases in the ICGC-OVAU dataset, and for the only applicable case “mRNA + clinical” in the Caldas-BC dataset (miRNA expression data not available). For comparisons under the same case, all hypothesis tests of median difference returned *p*-value ≤ 0.0001 (see Fig. 3).

We also applied modified-SALMON (LFSurv with eigengenes/eigen-miRNAs and demographic/clinical variables as inputs) to the non-TCGA datasets. When having same input features, modified-SALMON yielded significantly better performance than CoxPH-ENet, RSF, XGB-AFT, and XGB-CoxPH (*p*-value ≤ 0.001 for all tests, see Supplementary Fig. 3). This finding might partially support the application of multi-layer perceptron models in survival analysis. In Caldas-BC dataset, modified-SALMON yielded significantly higher C-index than AUTOSurv (median C-index 0.839 vs 0.780, *p*-value < 0.001). Note that for the two TCGA datasets, modified-SALMON also gained comparable prediction performance compared to AUTOSurv in the “mRNA + clinical” case, suggesting that “WGCNA + PCA” can be a good way to lower the dimension of gene expression data. For the “miRNA + clinical” case, however, AUTOSurv performed significantly better than modified-SALMON in all three available datasets (TCGA-BRCA, TCGA-OV, and ICGC-OVAU, *p*-value < 0.05 for all comparisons). This result shows that VAE is likely more generalizable than “WGCNA + PCA” approach in omics-data-dimension reduction. Moreover, for ICGC-OVAU dataset where the “mRNA + miRNA + clinical” case is applicable, AUTOSurv achieved higher C-index than the single omics scenarios (“mRNA + miRNA + clinical” vs “mRNA + clinical”: median C-index 0.735 vs 0.727, *p*-value = 0.093; “mRNA + miRNA + clinical” vs “miRNA + clinical”: median C-index 0.735 vs 0.711, *p*-value = 0.003), as well as versus modified-SALMON in “mRNA + miRNA + clinical” (median C-index 0.735 vs 0.691, *p*-value < 0.001), further demonstrating the strength of AUTOSurv in multi-omics integration for gene/miRNA expression data. Modified-SALMON on the other hand did not show significant improvement in prediction when both omics types were considered (“mRNA + miRNA + clinical” vs “mRNA + clinical”: median C-index 0.691 vs 0.684, *p*-value = 0.670; “mRNA + miRNA + clinical” vs “miRNA + clinical”: median C-index 0.691 vs 0.695, *p*-value = 0.788), again a possible outcome of the information overlap that we mentioned earlier.

LFSurv with only demographic/clinical variables as input was also fitted for the non-TCGA datasets. AUTOSurv (with omics data as part of its input) outperformed LFSurv [clincial only] approach for both datasets (ICGC-OVAU, AUTOSurv [“mRNA + miRNA + clinical”] vs LFSurv [clinical only]: median C-index 0.735 vs 0.685, *p*-value < 0.001; Caldas-BC, AUTOSurv [“mRNA + clinical”] vs LFSurv [clinical only]: median C-index 0.780 vs 0.729, *p*-value = 0.002), showing again the role of omics information in boosting model performance for prognosis prediction tasks in ovarian cancer and breast cancer studies.

Finally, we fitted the ML models on the TCGA-BRCA and the TCGA-OV datasets as well to assess their performance on bigger sample sizes. The results are illustrated in Supplementary Fig. 4. We see that on the TCGA datasets, AUTOSurv still outperformed the ML approaches.

### Risk-group prediction and DeepSHAP interpretation

**Fig. 4:**
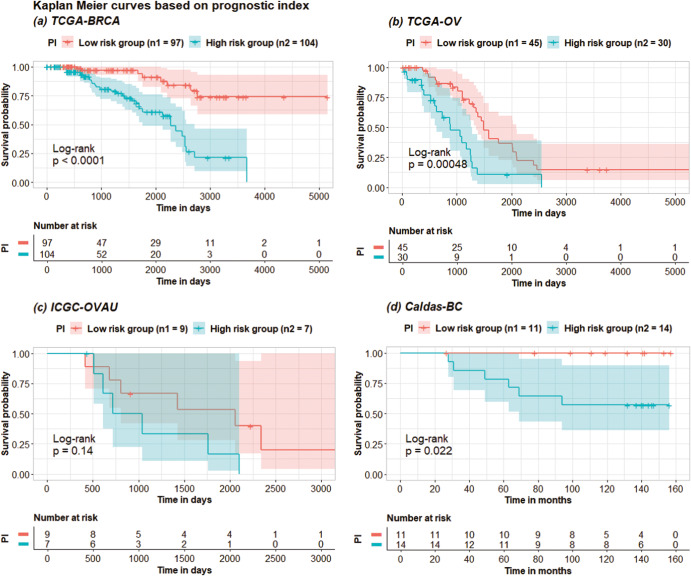
Kaplan-Meier (KM) curves for different risk groups. KM curves for the high-risk (${\rm{PI}} \,>\, {{\rm{PI}}}_{{\rm{med}}}$) and low-risk (${\rm{PI}}\le {{\rm{PI}}}_{{\rm{med}}}$) patient groups in the testing set of: **a** TCGA-BRCA dataset; **b** TCGA-OV dataset; **c** ICGC-OVAU dataset; **d** Caldas-BC dataset. ${{\rm{PI}}}_{{\rm{med}}}$ for each dataset is derived from the corresponding tuning set patients.

For each dataset, the AUTOSurv model that yielded the highest testing C-index in the “mRNA + miRNA + clinical” case was used as the final model to predict patients’ risk levels and identify important features via DeepSHAP. We first applied AUTOSurv on the tuning set and saved the median prognostic index (${{PI}}_{{med}}$) among the tuning set patients. We then applied AUTOSurv on the testing set, and patients with predicted *PI* > ${{PI}}_{{med}}$ were assigned to the high-risk group. Otherwise, they were assigned to the low-risk group. For the TCGA-BRCA, the TCGA-OV, and the Caldas-BC datasets, Fig. 4 shows significant differences between Kaplan–Meier (KM) curves of the two predicted risk groups (Log-rank test *p*-value < 0.05). Note that the ICGC-OVAU dataset has a very small testing set sample size (16 samples in total, 9 patients assigned to the low-risk group and 7 assigned to the high-risk group), which potentially explains the wide 95% confidence intervals of the KM curves. For the TCGA-BRCA and the TCGA-OV datasets, we have also produced KM curves based on age groups (i.e., by first quantile, median, and third quantile) and disease stages for the testing set patients (see Supplementary Fig. 5). On both datasets, ${{PI}}_{{med}}$-guided divisions showed greater difference in survival outcome between the two risk groups compared to age-group-guided divisions in terms of log-rank test p-values. On the TCGA-OV dataset, ${{PI}}_{{med}}$-guided division also outperformed all disease-stage-guided divisions. On the TCGA-BRCA dataset, although the ‘stage IV vs stages I - III’ division yielded log-rank test *p*-value < 0.0001, this finding is rather trivial since stage IV is already the latest stage and we only have 7 patients in this category. On the other hand, ${{PI}}_{{med}}$-guided division can give more plausible risk-group predictions to patients in earlier stages of breast cancer (lower log-rank test *p*-value than other disease-stage-guided divisions). Moreover, the age group and disease stage information used for those additional KM curves are directly from the testing set patients, yet ${{PI}}_{{med}}$ was derived from the tuning set data while treating the testing set as ‘unseen data’. This further demonstrates the capability of AUTOSurv in providing generalizable predictions.

**Fig. 5:**
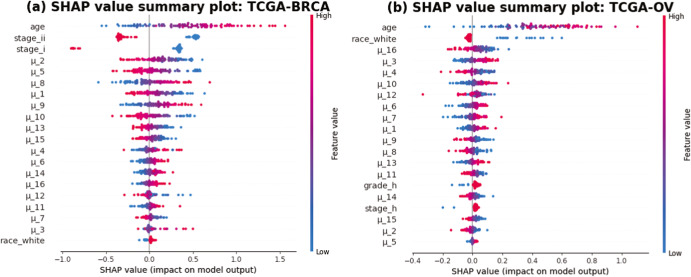
SHAP value summary plots. Summary plot of SHAP values for 100 randomly sampled high-risk patients from **a** TCGA-BRCA dataset and **b** TCGA-OV dataset. Y-axis lists the input features of the LFSurv network, ranked by their overall contribution scores. X-axis shows the SHAP values of the dots. Each dot represents a patient, the colors indicate their corresponding feature values, with red representing high values and blue representing low values. For the TCGA-OV dataset, we have stage_h = 1 for patients with stage III or IV (higher stages) ovarian-cancer, patients with lower disease stages have stage_h = 0. Patients with histological grades G3 or G4 (higher grades) have grade_h = 1, patients in lower histological grades have grade_h = 0.

We conducted an interpretation study via DeepSHAP (see the “Methods” section) based on tuning set data. Here we present in detail the interpretation results for the TCGA-BRCA and TCGA-OV datasets. Results for the non-TCGA datasets can also be found on our Github website https://github.com/jianglindong93/AUTOSurv. Following the procedures mentioned in^26^, the SHAP values (contribution scores in our setting) of each LFSurv input feature were calculated for 100 randomly sampled high-risk-group patients. We present in Fig. 5 the SHAP value summary plots. Features on the Y-axis are sorted in descending order based on their overall contribution scores (averaged absolute SHAP values, see the “Methods” section). Within each row, each dot represents a patient, the color of the dot indicates its feature value, with red and blue corresponding to high and low values on the spectrum, respectively. The X-axis specifies the intensity and direction of the SHAP values. As an example, for the feature ‘age’, the dots become redder as they go further along the positive side of the X-axis, which means that a higher age will contribute to a higher predicted *PI* value, hence higher risk of death. Moreover, we see that for both datasets, the top features with the highest overall contribution scores are clinical variables (age and clinical stage in the TCGA-BRCA dataset; age and race in the TCGA-OV dataset). This shows that clinical variables can play important roles in survival analysis and should not be ignored even when omics data are available.

For each of the datasets, a total of 16 latent features (i.e., ${\{{\mu }_{i}\}}_{i=1,\ldots ,16}$) were extracted from the two omics data types and input into LFSurv. The number of latent features to extract (i.e., number of latent features in the bottleneck layer) was tuned as a hyperparameter. We chose 16 because it was in the best set of hyperparameters that yielded smallest reconstruction loss for KL-PMVAE (for both datasets). The numbers of latent features we tuned across were summarized in Supplementary Table 4. To identify genes/miRNAs that contributed most to the important latent features, we calculated KL-PMVAE input factor (gene/miRNA) contribution scores for each of the top 6 latent features in Fig. 5 (i.e., ${\mu }_{2}$, ${\mu }_{5}$, ${\mu }_{8}$, ${\mu }_{1}$, ${\mu }_{9}$, ${\mu }_{10}$ for TCGA-BRCA; ${\mu }_{16}$, ${\mu }_{3}$, ${\mu }_{4}$, ${\mu }_{10}$, ${\mu }_{12}$, ${\mu }_{6}$ for TCGA-OV). For each dataset, if a gene or miRNA had high contribution scores (i.e., top 10 among all input factors) for more than one latent feature, we identified it as a Key Input Factor (KIF). In Supplementary Fig. 6, we present the identified KIFs for TCGA-BRCA and TCGA-OV with their frequencies as top 10 most contributing factors of latent features.

For TCGA-BRCA dataset, the identified KIFs included 11 genes, all of which were found to be associated with breast cancer in existing studies (see Supplementary Table 5 for more details). For example, the study^29^ found that CDC20 knockdown inhibited the migration of metastatic MDA-MB-231 breast cancer cell line. Recent studies also demonstrated that FABP4 promotes obesity-associated breast cancer development^30^. For PSMB9, the study^31^ found that PSMB9 was overexpressed in breast cancer cells. For PLIN1, the study^32^ found that its mRNA expression is significantly downregulated in human breast cancer.

For TCGA-OV dataset, we identified 5 genes and 1 miRNA as KIFs (Supplementary Fig. 6b). Four of these factors (FGF18, HERC5, RPS27A, and hsa-miR-202) were found to be associated with ovarian cancer in previous literatures^33–36^. Overexpression of FGF18 was identified as a predictive marker for poor clinical outcomes in patients with advanced stage, high-grade serous ovarian cancer by ref. ^33^. HERC5 was found to have increased expression levels in topotecan-resistant ovarian cancer cell lines by^34^. The study^35^ identified genes with survival-related alternative splicing events in ovarian cancer, and RPS27A was one of the hub genes in the gene interaction network. For hsa-miR-202^36^, found that miR-202-5p was downregulated in ovarian cancer and verified the role of miR-202-5p in suppressing cell proliferation, migration, and invasion in ovarian cancer. Moreover, we obtained the predicted target genes of miR-202-3p from *miRDB*^37^ and performed gene set functional enrichment analysis on these target genes via *ToppGene Suite*^38^. We found that the Gene Ontology (GO) terms for extracellular matrix organization (GO:0030198, FDR adjusted *p*-value = 6.16E-07) and extracellular structure organization (GO:0043062, FDR adjusted *p*-value = 6.16E-07) were significantly enriched. Interestingly, the paper^39^ pointed out that extracellular matrix (ECM) dysregulation can occur during ovarian tumorigenesis, and it plays a role in tumor progression.

Surprisingly, for both datasets, we found that the associations between the identified key genes’ expression levels and the survival outcomes cannot be directly inferred via simple survival analysis. We created dummy variables according to the median expression values of the key genes and fitted univariate CoxPH models using these variables, however, none of the genes reached significance after multiple testing correction (see Supplementary Table 6). It could be due to the insufficient power, given the low sample size or event rate in the TCGA datasets (TCGA-BRCA dataset has a low event rate [16.54%] and TCGA-OV dataset has a small sample size [355]). We did a post-hoc power analysis at significance level of 0.05. For TCGA-BRCA, we have 80% power to detect a gene having hazard ratio (HR) > 1.23 or HR < 0.81. For TCGA-OV, we have 80% power to detect a gene having HR > 1.21 or HR < 0.82. Many of the genes did not fall in these detectable intervals (5 out of 11 genes in TCGA-BRCA; 4 out of 5 genes in TCGA-OV, see Supplementary Table 6). Another reason might be due to the complex hidden mechanism underlying cancer progression, which makes it difficult for univariate/linear models to capture the associations with a single gene. DeepSHAP on the other hand, is not constrained by statistical assumptions. Moreover, it takes non-linear relationships into account when backpropagating through the DNN and considers the expression levels of all other genes when calculating the contribution scores of a single gene. Therefore, DeepSHAP has the potential to locate important genes that are undetectable in univariate survival analysis. However, at this stage of our interpretation process, we can only ‘locate’ the key genes. To further uncover their prognostic relevance (e.g., the directions of the associations), we may need to delve deeper into harnessing the SHAP values (e.g., combine the contributions from a specific gene to different latent features and weigh the contribution from each latent feature to the final prediction), which is a potential direction of our future study.

We made full use of the interpretation-friendly design of our DNN (i.e., pathway-information-guided node connection, see the “Methods” section for more details) and applied the same procedure to concurrently identify the Key Pathway Factors (KPFs) for the top 6 latent features. The identified pathways and their frequencies are illustrated in Supplementary Fig. 7. For TCGA-BRCA, evidence of association with breast cancer or cancer in general can be found for all the identified pathways in previous literatures (see Supplementary Table 5 for list of references). The pathway with frequency three in Supplementary Fig. 7a, R-HSA-163560, is triglyceride catabolism. According to ref. ^40^, triglyceride was found to be significantly elevated among breast cancer patients compared to controls, and their study suggested that higher levels of triglyceride may play important role in carcinogenesis. Using DeepSHAP, we were able to identify KIFs that contributed most to KPFs. For the KPF: R-HSA-163560 in the pathway layer, the top two contributing factors were KIFs: FABP4 and PLIN1. According to ref. ^41^, FABP4 was positively associated with triglycerides in breast cancer patients. The study^32^ noted that PLIN1 plays a distinct role in regulating both triglyceride storage and lipolysis in adipocytes, and that reduced expression of PLIN1 could be an independent predictor of overall survival for breast cancer patients.

For the TCGA-OV dataset, evidence of association with ovarian cancer can be found in previous literatures for six of the identified pathways (see Supplementary Table 5 for list of references). The two pathways with frequency three in Supplementary Fig. 7b are R-HSA-168928 and R-HSA-72163, which correspond to RIG-I/MDA5 mediated induction of IFN-alpha/beta, and mRNA splicing, respectively. According to ref. ^42^, it has been reported that IFN-alpha specifically targets a subset of ovarian cancer cells that have stem-like properties. The study^43^ found that high expression of RIG-I is associated with poor clinical outcomes in ovarian cancer. The study^44^ conducted prognosis prediction for ovarian-cancer patients based on alternative splicing (AS) events and suggested AS sites as potential targets for ovarian-cancer treatment. Pathway R-HSA-168928 includes three of the KIFs in Supplementary Fig. 6b (HERC5, RPS27A, and UBA52). For the node corresponding to R-HSA-168928 in the pathway layer of KL-PMVAE, HERC5 is the input factor that had the highest contribution score with respect to the difference in its node values between high- and low-risk groups.

Overall, the results suggest that DeepSHAP has the potential to reveal hidden mechanisms underlying breast and ovarian-cancer survival and may provide support and guidance for future molecular-level investigations.

### External-cross-dataset validation

**Fig. 6:**
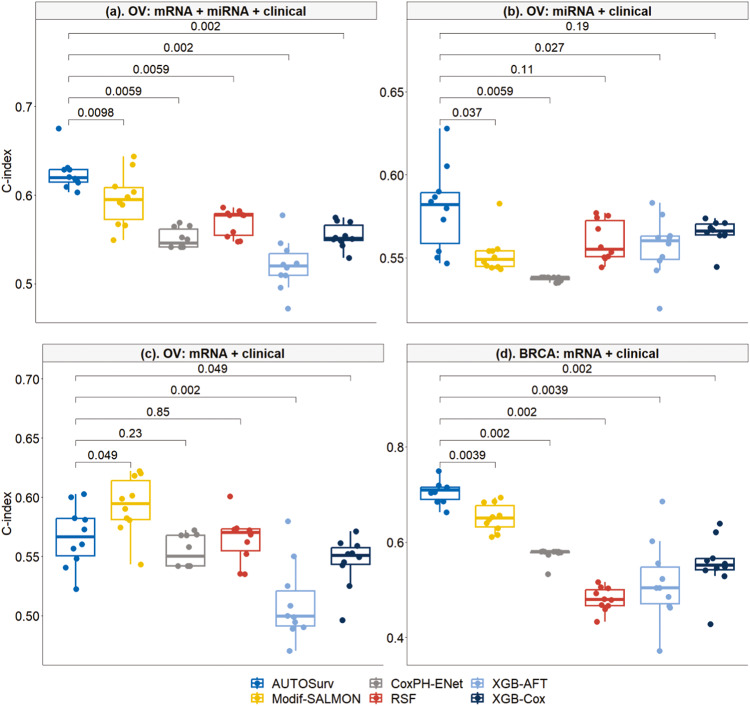
Performance of different models when trained on TCGA datasets and tested on non-TCGA datasets. OV: Models trained on TCGA-OV dataset and tested on ICGC-OVAU dataset; BRCA: Models trained on TCGA-BRCA dataset and tested on Caldas-BC dataset. **a** the “mRNA + miRNA + clinical” case in OV; **b** the “miRNA + clinical” case in OV; **c** the “mRNA + clinical” case in OV; **d** the “mRNA + clinical” case in BRCA. The *p*-value from two-sided Wilcoxon signed-rank test (i.e., null hypothesis ${{\rm{H}}}_{0}$: median difference is equal to 0; versus alternative hypothesis ${{\rm{H}}}_{{\rm{A}}}$: median difference is not 0) is displayed between boxes.

To compare the generalizability of the models across datasets, we trained and tuned the models on the two TCGA cancer datasets respectively and applied the trained models to the independent external non-TCGA datasets correspondingly for external validation. For example, train/tune on TCGA-OV and test on ICGC-OVAU; train/tune on TCGA-BRCA and test on Caldas-BC. AUTOSurv and other machine learning methods were all tested. Similar to the framework evaluation analysis mentioned above, “WGCNA + PCA” procedure was implemented to reduce the dimension of gene/miRNA expression data for the machine learning approaches and the modified-SALMON method. The results were summarized in Fig. 6.

When trained on TCGA-OV dataset and tested on ICGC-OVAU dataset, AUTOSurv outperformed all other methods when both omics types were used as input (see “OV: mRNA + miRNA + clinical” in Fig. 6). For the single omics cases, AUTOSurv achieved comparable or higher C-index compared to the machine learning methods (see “OV: miRNA + clinical” and “OV: mRNA + clinical” in Fig. 6). The modified-SALMON method performed significantly better than AUTOSurv in the “mRNA + clinical” case of OV (AUTOSurv vs Modif-SALMON median C-index: 0.567 vs 0.595, *p*-value = 0.049), while AUTOSurv yielded significantly higher C-index in the “miRNA + clinical” case (AUTOSurv vs Modif-SALMON median C-index: 0.582 vs 0.549, *p*-value = 0.037). Moreover, in OV, the performance of modified-SALMON did not differ between the “mRNA + clinical” case and the “mRNA + miRNA + clinical” case (median C-index = 0.595 for both cases, *p*-value = 0.959) while AUTOSurv showed significantly improved performance when both omics types were included as input compared to single omics cases (median C-index, “mRNA + miRNA + clinical” vs “mRNA + clinical”: 0.619 vs 0.567, *p*-value = 0.002; “mRNA + miRNA + clinical” vs “miRNA + clinical”: 0.619 vs 0.582, *p*-value = 0.004), which is consistent with our findings in the previous sections. When trained on TCGA-BRCA dataset and tested on Caldas-BC dataset with gene expression data and clinical variables as input, AUTOSurv gained best performance compared to all other methods (see “BRCA: mRNA + clinical” in Fig. 6). Generally, when trained on TCGA datasets and tested on external non-TCGA datasets, AUTOSurv showed highest across-dataset-generalizability compared to other approaches. Furthermore, KL-PMVAE of AUTOSurv yielded better overall performance than “WGCNA + PCA” in omics-data-dimension reduction, and AUTOSurv maintained its high efficiency in integrating multi-omics features.

For both cancer types, AUTOSurv with omics data as part of its input gained significantly higher C-index than LFSurv with only demographic/clinical variables as input (OV, AUTOSurv [“mRNA + miRNA + clinical”] vs LFSurv [clinical only]: median C-index 0.619 vs 0.54, *p*-value = 0.002; BRCA, AUTOSurv [“mRNA + clinical”] vs LFSurv [clinical only]: median C-index 0.709 vs 0.672, *p*-value = 0.004). This indicates that omics information can help improve model performance even in cross-dataset scenarios.

**Fig. 7:**
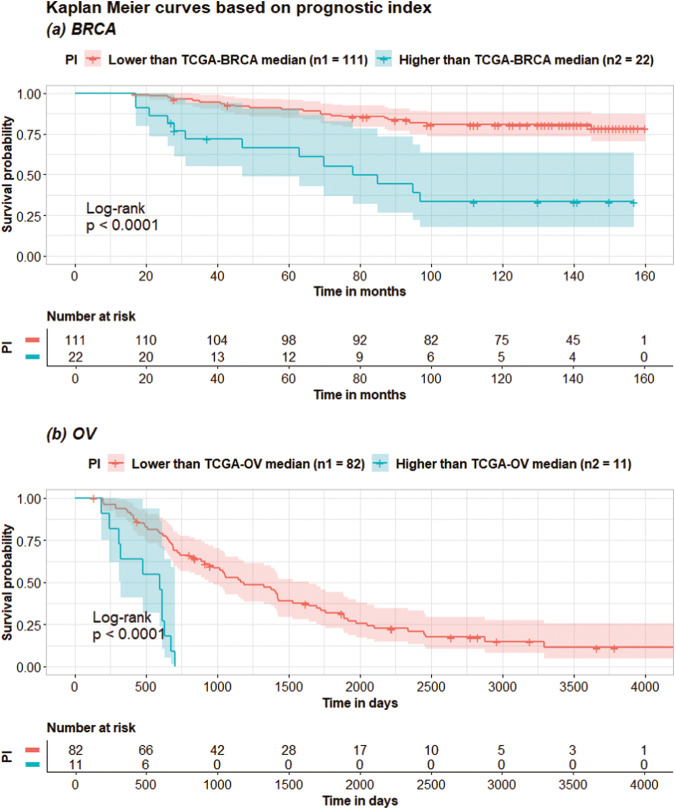
Kaplan-Meier (KM) curves for different risk groups in the non-TCGA datasets during external-cross-dataset validation. KM curves for the high-risk (${\rm{PI}} > {{\rm{PI}}}_{{\rm{med}}}^{{\rm{TCGA}}}$) and low-risk (${\rm{PI}}\le {{\rm{PI}}}_{{\rm{med}}}^{{\rm{TCGA}}}$) patient groups in the non-TCGA datasets. **a** BRCA: KM curves for patients in the Caldas-BC dataset, with ${{\rm{PI}}}_{{\rm{med}}}^{{\rm{TCGA}}}$ derived from the TCGA-BRCA dataset; **b** OV: KM curves for patients in the ICGC-OVAU dataset, with ${{\rm{PI}}}_{{\rm{med}}}^{{\rm{TCGA}}}$ derived from the TCGA-OV dataset.

Additionally, we saved the best performing AUTOSurv model in the “mRNA + miRNA + clinical” case of OV and the “mRNA + clinical” case of BRCA, respectively. Prognostic indices of all patients were calculated using the saved models. For each TCGA dataset, we denote the median prognostic index among its patients as ${{PI}}_{{med}}^{{TCGA}}$, and patients in its corresponding external validation dataset will be assigned to high- and low-risk groups based on ${{PI}}_{{med}}^{{TCGA}}$ (high-risk group if ${PI} \,>\, {{PI}}_{{med}}^{{TCGA}}$, low-risk group if ${PI}\le {{PI}}_{{med}}^{{TCGA}}$). KM curves for the two risk groups in the external validation datasets were illustrated in Fig. 7. For both cancer types, we can see from Fig. 7 that AUTOSurv-derived ${{PI}}_{{med}}^{{TCGA}}$ from the TCGA datasets can guide highly distinguishable risk group divisions on the non-TCGA datasets as well. This finding further implies AUTOSurv’s cross-dataset generalizability.

## Discussion

AUTOSurv is a deep learning model consisting of a specially designed upstream KL-PMVAE network that extracts low-dimensional latent features from high-dimensional omics data; and a downstream multi-layer perceptron LFSurv that receives the extracted latent features and the demographic/clinical variables as combined input and calculates a predicted prognostic index (*PI*) for each patient. We applied AUTOSurv in different scenarios. It achieved the highest C-index when gene expression, miRNA expression, and clinical data were all used. At developmental stage with TCGA datasets, the highest C-index from AUTOSurv was achieved in the case where the “entangle” integration strategy was combined with the KL-annealing learning scheme. Moreover, although the incorporation of omics data improved model performance (in developmental stage: median C-index 0.714 vs 0.749 for TCGA-BRCA dataset, median C-index 0.623 vs 0.629 for TCGA-OV dataset; in external-cross-dataset validation: median C-index 0.54 vs 0.619 for ICGC-OVAU dataset, median C-index 0.672 vs 0.709 for Caldas-BC dataset), during our interpretation analysis some clinical variables (e.g., age, disease stage, race) in the TCGA datasets were assigned the highest contribution scores by DeepSHAP among all input features of LFSurv. This suggests that clinical variables are vitally important for survival analysis and should not be ignored regardless of access to other types of modalities. Nevertheless, from Fig. 4 and Supplementary Fig. 5, we see that AUTOSurv-derived *PI* conferred more plausible risk-group predictions than age and disease stage, which implies the potential of omics-data-infused deep learning models in assisting clinical diagnosis and treatment. For example, the estimated *PI* values from AUTOSurv can be combined with age and disease stage information to build a more accurate treatment recommendation system. Finally, during framework evaluation, AUTOSurv outperformed other widely applied machine learning approaches (i.e., CoxPH-ENet, RSF, XGB-CoxPH, and XGB-AFT) in all cases on two non-TCGA datasets. Also, compared to the machine learning approaches, AUTOSurv showed better overall performance and maintained highly effective in multi-omics integration when trained/tuned on TCGA datasets and tested on non-TCGA datasets. These results show the strength of deep neural networks in handling complex data structures and the high efficiency of AUTOSurv in integrating gene expression/miRNA expression data. Although we only studied breast cancer and ovarian-cancer data in this paper, our approach can be directly implemented to perform prognosis prediction and result interpretation for other cancer types.

By applying DeepSHAP to TCGA-dataset-trained-AUTOSurv, we identified genes, miRNA, and pathways that were important for distinguishing predicted high- and low-risk-group patients, most of which were found to be associated with breast/ovarian cancer or cancer in general in previous studies. This is reassuring as it implies that it is indeed biologically relevant information rather than random events that is guiding the model predictions. By virtue of the interpretation-friendly design of KL-PMVAE, we linked the key pathways with the key genes. This showed that “AUTOSurv + DeepSHAP” could help us (1) identify potential biomarkers for cancer prognosis and (2) reveal which pathways will provide insight into hidden mechanisms. Even so, DeepSHAP can yield inconsistent results and the same model does not always assign identical importance to the input features at different DeepSHAP implementations^45^. This inconsistency was more frequently observed when applying DeepSHAP to AUTOSurv models trained on small sample sizes (i.e., ICGC-OVAU and Caldas-BC datasets, although RPS27A was found to be a common KIF for both TCGA-OV and ICGC-OVAU datasets). Therefore, the procedure for selecting key features can be further improved and standardized to obtain more reliable and robust interpretations. For instance, we could utilize the SHAP values (i.e., contribution scores, see the “Methods” section) generated by DeepSHAP as quantitative measures instead of focusing only on their rankings. We could also consider the +/- signs of the SHAP values to make our interpretation more informative. The way of incorporating those signs, however, needs to be selected with caution so the positive and negative contribution scores won’t simply cancel each other out hence undermine the importance of the features.

In this study, we assumed that the interactive crosstalk (facilitated by presumably overlapping information) between gene expression and miRNA expression data enabled more efficient multi-omics integration. The view-specific information (i.e., here we consider each type of omics data as a different view of the samples^13^), however, can also be important especially when the overlapping information between different types of omics data is trivial. Moreover, disentangling view-specific and view-shared aspects of latent features may make the VAE more interpretable. Models like Deep Probabilistic CCA (DPCCA)^46^ might be useful for such disentanglement tasks and combining this approach with our KL-PMVAE for better multi-omics integration and feature extraction is a potential future direction of study. Integration of multiple modalities is another relevant topic, and some studies have attempted to include whole slide image data^10,12^ as one extra input modality for prognosis prediction. For AUTOSurv we concatenated the latent features (extracted from omics data) and the clinical variables directly in the input layer of LFSurv. This approach is straightforward, but our results suggest that concatenation may not be the best way to handle complex relationships between different modalities. We expect more delicate model designs to be developed for multimodal representation learning in our future pursuits.

There are several directions that AUTOSurv can be improved in the future. Firstly, although AUTOSurv achieved better performance in many cases compared to other DL models (i.e., CoxPASNet^6^, OmiVAE^15^, SALMON^11^, with modifications made to better suit our purpose), in some cases, Surv-OmiVAE outperformed AUTOSurv. For example, in TCGA-BRCA dataset, when miRNA expression data and clinical variables were used as input, Surv-OmiVAE achieved median C-index 0.756 vs 0.738 from AUTOSurv. This implies that proper modifications to AUTOSurv allowing task-oriented feature extraction may be a good starting point to developing a more advanced model. Secondly, in this study we excluded many genes to facilitate the “pathway-masking” design of KL-PMVAE (see the “Methods” section for more details), which could cause information loss. Improvements in the pre-filtering process will be another focus in future studies. For instance, we could combine multiple pathway databases and/or change the selection criteria for the pathway nodes (e.g., only exclude pathways that contain fewer than 10 genes or greater than 500 genes in our dataset) to expand genome coverage. Thirdly, although AUTOSurv achieved the best performance compared to other approaches, its predictive performance on the ovarian-cancer cohorts still lacks clinical meaningfulness. One biggest challenge is that the sample sizes of these cohorts are even smaller than the breast cancer cohorts, which can lead to insufficient training data for AUTOSurv to learn from. In future study, we could apply transfer learning techniques^47^ to borrow strength from models that are trained on larger datasets from other cancer types or isolated single-omics OV data. Furthermore, most of the patients in the TCGA-OV dataset and all patients in the ICGC-OVAU dataset are in advanced stages of ovarian cancer while most patients in the breast cancer datasets have early-stage breast cancer, our future study could pay more attention to this difference when developing ovarian-cancer-specialized prediction models. Lastly, the downstream LFSurv section of AUTOSurv is a Cox Proportional Hazard network, and studies have tried to overcome the proportional hazard constraint to yield more realistic predictions^12,14,48^. We could also adjust the learning objectives of AUTOSurv accordingly to model time-varying effects of the input features and/or to learn patient-specific survival distributions^14,48^.

## Methods

### Overview of AUTOSurv

**Fig. 8:**
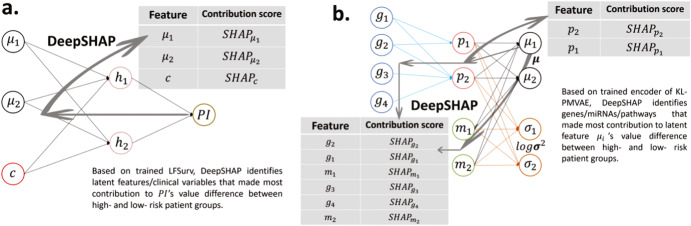
Illustration for DeepSHAP implementations. DeepSHAP implementations to identify **a** latent features/clinical variables that contribute most to the difference in ${\rm{PI}}$ between high- and low-risk groups, and **b** pathways/genes/miRNAs that contribute most to the difference in latent-feature values (between high- and low-risk groups) for the most important latent features found in (**a**). SHAP Shapley Additive Explanations (SHAP) value (i.e., the contribution score in our setting).

AUTOSurv is a deep learning model for cancer prognosis prediction combining multi-omics and demographic/clinical data. There are three major parts to our framework: (1) pathway information incorporated multi-omics variational autoencoder (VAE) with KL-annealing learning strategy^49^ (KL-PMVAE) for efficient multi-omics integration and latent-feature extraction; (2) latent-feature-fed survival network (LFSurv) integrates latent features extracted by the VAE model with demographic/clinical variables and conducts final prognosis prediction; and (3) DeepSHAP^25,26^ interpretation approach applied to the trained AUTOSurv model (KL-PMVAE plus LFSurv) assigns importance scores to input features and identifies the features that make important contribution in distinguishing between the high- and low-risk patients. The workflow of AUTOSurv is illustrated in Fig. 1. The implementations of DeepSHAP are illustrated in Fig. 8.

### Data and preprocessing

For model development, we collected survival outcomes (overall survival time and censoring status), demographic/clinical records (e.g., age, disease stage, race), and gene and miRNA expression data for 1,058 female patients with stage I - IV breast cancer, and for 355 female patients with stage I - IV ovarian cancer from the Genomic Data Commons (GDC) Breast Cancer (BRCA) cohort and Ovarian Cancer (OV) cohort of The Cancer Genome Atlas (TCGA) program, respectively. Data were downloaded from UCSC Xena data portal (https://xenabrowser.net/datapages/)^50^ on October 30th, 2021. Demographics of the patients are summarized in Supplementary Table 1. There are 175 and 222 observed deaths among the patients for the TCGA-BRCA dataset and TCGA-OV dataset, respectively. For both TCGA-BRCA and TCGA-OV datasets on the UCSC Xena portal, the gene expression data contain ${\log }_{2}$-transformed fragments per kilobase of transcript per million mapped reads (FPKM), and the miRNA expression data contain ${\log }_{2}$-transformed normalized counts in reads-per-million-miRNA-mapped (RPM). Although mRNA and miRNA expression data both come from transcriptome, here we treat them as two omics because gene regulation by miRNA is part of epigenetic mechanisms^51^.

For performance evaluation and comparison with other existing survival models, we collected survival outcomes, demographic/clinical records, and gene and miRNA expression data for 133 female patients with stage I - III breast cancer, and for 93 female patients with stage III - IV ovarian cancer from the UCSC Xena Caldas 2007 Breast Cancer cohort^52^ (Caldas-BC) and International Cancer Genome Consortium (ICGC) Ovarian Cancer – Australian (OVAU) cohort (https://dcc.icgc.org/releases/current/Projects/OV-AU), respectively (Caldas-BC cohort does not have miRNA expression data, neither of the cohorts have race information recorded). Demographics of the patients are summarized in Supplementary Table 1. For the Caldas-BC cohort, there are 35 deaths from breast cancer observed. For the ICGC-OVAU cohort, 74 deaths were observed.

We studied genes on autosomes and the X chromosome. For both BRCA and OV datasets, we randomly extracted 20% of patients as testing set which was not involved in any of the model training/tuning procedures during our experiments. The remaining 80% of patients were treated as tuning set and further divided into training and validation sets with the ratio of 4:1. In each of the training/validation/testing sets, the gene/miRNA expression data were rescaled to the range of 0 to 1 using min-max normalization (Eq. 1) to fit the input requirement of our VAE model^15^. To denoise the two omics data types, we followed the filtering procedure described in^53^ and excluded genes/miRNAs with variance of < 0.02 in the min-max normalized tuning set. A summary of the omics features before and after preprocessing can be found in Supplementary Table 2. The min-max normalization process is summarized as follows:

1 $${v}_{{minmax}}^{(i)}=\frac{{v}^{(i)}-{v}_{min }}{{v}_{max }-{v}_{min }}$$

where ${v}^{(i)}$ and ${v}_{{minmax}}^{(i)}$ are the expression data values for feature *v* in patient *i* before and after min-max normalization. ${v}_{max }$ (${v}_{min }$) is the maximum (minimum) value of *v* across all patients in the dataset considered.

### Pathway-mask guided variational autoencoder

We built a VAE model to compute low-dimensional latent variables ${{\boldsymbol{z}}}^{(i)}\in {{\mathbb{R}}}^{d}$ from high-dimensional omics data ${\{{{\boldsymbol{x}}}^{(i)}\}}_{i=1,\ldots ,N},{{\boldsymbol{x}}}^{(i)}\in {{\mathbb{R}}}^{p}$ (*N* is the number of patients; *p* is the number of input features [e.g., number of genes]; *d* is the number of latent variables computed from the input data, and $p\gg d$), which can reduce the risk of overfitting in the prognosis prediction task. Unlike classic autoencoder (AE) models, VAE learns a distribution estimate instead of a point estimate for the low-dimensional latent variables ***z***^54^, which can potentially increase the efficiency of the information extraction process and generate “disentangled” latent representations of the input features. This “disentanglement” allows qualitatively different information to be encoded into distinct latent variables, which could contribute to a more interpretable VAE model^55,56^.

As illustrated in Fig. 1c, the encoder part of KL-PMVAE consists of a gene layer (each node represents a gene), a pathway layer (each node represents a pathway), and a miRNA layer (each node represents a miRNA). Reactome^57^ pathway information was obtained from the online resource Database for Annotation, Visualization, and Integrated Discovery (DAVID)^58^ (by the time we collected the pathway information, DAVID adopted Reactome database Version 78 in its knowledgebase [https://david.ncifcrf.gov/content.jsp?file=update.html]). We chose Reactome pathways because they have the widest coverage for the gene list we submitted. Sparse connections were forced between the gene layer and the pathway layer, where a gene node is connected to a pathway node only if that gene belongs to that specific pathway. According to ref. ^59^, small pathways can be redundant with larger pathways and large pathways can be overly general, both of which can hamper the interpretability of the model. We can rephrase this in the sense of deep neural networks. On the one hand, pathway nodes that take information from too many gene nodes can complicate the interpretation process, especially when we are trying to identify the most important genes for model prediction. The pathway itself can be overly general from a biological perspective, and its relationship with disease outcome might be uninformative. Moreover, including “overly general” pathway nodes may result in too many trainable parameters in the deep neural network and make the model prone to overfitting. On the other hand, since we have thousands of genes in total, a pathway node connects to very few gene nodes may only encode trivial information, and detection of important features can be difficult when they are surrounded by noisy features. Therefore, we excluded pathways that contain fewer than 15 genes and pathways with more than 300 genes in our datasets. Note that there is no gold standard for deciding “too many” or “too few” gene-node connections for the pathway nodes; different selection criteria may result in varied model performance, but the comparison between different selection threshold is out of the scope of this study. Only genes belonging to at least one of the remaining pathways were kept. The same sparse connection pattern was kept between the last two layers in the gene part of the decoder (Fig. 1c). This pathway-mask design is inspired by the mask-matrix-forced connections introduced by ref. ^6^. It not only incorporates prior biological knowledge into the network but also reduces the number of trainable parameters compared to a fully connected design and hence yields a lower risk of overfitting.

For multi-omics integration, there is a noticeable difference between the dimensionalities of the two omics data types (for TCGA-BRCA, TCGA-OV, and ICGC-OVAU datasets, the number of genes is more than four times that of miRNAs, Supplementary Table 2). To mitigate potential imbalance in model training/parameter learning, we concatenated the pathway layer, instead of the gene layer, with the miRNA layer (Fig. 1c). Because the pathway nodes contain forward-propagated-gene-node information, and the number of pathways is more comparable to the number of miRNAs. The concatenated features were then forward propagated to produce the means ***μ*** and log-variances $\log {{\boldsymbol{\sigma }}}^{2}$ for the latent variables ${\boldsymbol{z|x}}{\boldsymbol{ \sim }}N({\boldsymbol{\mu }},{{\boldsymbol{\sigma }}}^{2})$, where $N({\boldsymbol{\mu }},{{\boldsymbol{\sigma }}}^{2})$ is the estimated posterior distribution ${q}_{\phi }({\boldsymbol{z}}|{\boldsymbol{x}})$, $\phi$ is the set of learnable parameters in the encoder^15^. To sample from the distribution estimate, we applied the reparameterization trick:

2 $$\hat{{\boldsymbol{z}}}={\boldsymbol{\mu }}+{\boldsymbol{\sigma }}{\boldsymbol{\varepsilon }},{\boldsymbol{\varepsilon }} \sim N({\bf{0}},{\boldsymbol{I}})$$

which enables backpropagation for the VAE^54^. The decoder takes the sampled latent variable values $\hat{{\boldsymbol{z}}}$ as input and reconstructs the gene expression and miRNA expression data (i.e., ${\hat{{\boldsymbol{x}}}}_{{gene}}$ and ${\hat{{\boldsymbol{x}}}}_{{miRNA}}$ respectively). The loss function for our VAE model is as follows:

3 $$\begin{array}{l}{L}_{{VAE}}={BCE}\left({{\boldsymbol{x}}}_{{gene}},{\widehat{{\boldsymbol{x}}}}_{{gene}}\right)+{BCE}\left({{\boldsymbol{x}}}_{{miRNA}},{\widehat{{\boldsymbol{x}}}}_{{miRNA}}\right)\\\qquad\quad+\,\beta {D}_{{KL}}(N({\boldsymbol{\mu }},{{\boldsymbol{\sigma }}}^{2})\parallel N({\bf{0}},{\boldsymbol{I}}))+{\lambda }_{1}\parallel {{{\boldsymbol{\theta }}}_{1}{\boldsymbol{\parallel }}}_{2}\end{array}$$

where ${BCE}({\boldsymbol{x}},\hat{{\boldsymbol{x}}})$ is the binary cross-entropy between the input expression data ${\boldsymbol{x}}$ and the reconstructed expression data $\hat{{\boldsymbol{x}}}$. The term ${D}_{{KL}}(N({\boldsymbol{\mu }},{{\boldsymbol{\sigma }}}^{2}){||N}({\boldsymbol{0}},{\boldsymbol{I}}))$ is the KullbackLeibler (KL) divergence^60^ between the estimated posterior distribution $N({\boldsymbol{\mu }},{{\boldsymbol{\sigma }}}^{2})$ and the prior distribution $N({\boldsymbol{0}},{\boldsymbol{I}})$. The term $\parallel {{{\boldsymbol{\theta }}}_{1}{{\parallel }}}_{2}$ is the ${L}_{2}$-norm of the learnable parameters in KL-PMVAE and ${\lambda }_{1}$ is the regularization parameter that can be tuned to control the severity of the penalization. The value of *β* controls how much emphasis should be placed on the KL-divergence term of the loss function and is set to 1 in conventional VAE but will be changed gradually from 0 to 1 in a KL-annealing learning scheme as described below. When KL-divergence equals 0, the posterior equals an isotropic unit Gaussian regardless of the input features ***x***; therefore, the minimization of the KL-divergence term implies a limitation on the amount of information that can pass through the latent bottleneck (Fig. 1c). According to ref. ^56^, this constraint, combined with the pressure to minimize reconstruction loss, encourages the model to learn a more efficient representation of the data.

For model implementation, we applied batch normalization for all layers except for the latent bottleneck. Rectified linear units (ReLU), linear, and sigmoid activation functions were used for certain layers as illustrated in Fig. 1c.

### Latent-feature-fed survival network for prognosis prediction

To conduct survival analysis, we built a fully connected (FC) DL network as illustrated in Fig. 1d, which can be viewed as a shallower version of DeepSurv^20^. The extracted latent features from KL-PMVAE (i.e., means ***μ*** of the learned distribution estimate of the latent variables ***z***) were concatenated with the demographic/clinical variables (e.g., age, disease stage, race) and input into the network. After forward propagation through one hidden layer, the network outputs a prognostic index (*PI*)^6^ for each patient, which is the estimate of the log-risk function in a CoxPH model^20^. High *PI* indicates a poor prognosis and vice versa. Like DeepSurv, the objective function of this FC network is the average negative log-partial likelihood with ${L}_{2}$ regularization:

4 $$l\left({{\boldsymbol{\theta }}}_{2}\right)=-\frac{1}{{n}_{E=1}}\mathop{\sum}\nolimits _{i:{E}_{i}=1}({{PI}}_{i}-\log \mathop{\sum}\nolimits _{j\in R\left({T}_{i}\right)}{e}^{{{PI}}_{j}})+{\lambda }_{2}\parallel {{{\boldsymbol{\theta }}}_{2}{{\parallel }}}_{2}$$

where ${n}_{E=1}$ is the number of uncensored patients, and $R\left({T}_{i}\right)=\left\{i:{T}_{i}\ge t\right\}$ is the set of patients still at risk of failure at time *t*. The term $\parallel {{{\boldsymbol{\theta }}}_{2}{{\parallel }}}_{2}$ is the ${L}_{2}$-norm of the learnable parameters in LFSurv and ${\lambda }_{2}$ is the regularization parameter that can be tuned to control the severity of the penalization^6^.

Dropout was applied to prevent overfitting. Hyperbolic tangent (tanh) activation was applied to compute node values for the hidden layer and linear activation was applied to compute the *PI* value in the output layer. Because the linear combination of predictors in CoxPH does not contain a constant term^8^, the linear activation for the output layer has no bias term either.

### Prediction performance evaluation

C-index was used to measure model performance in prognosis prediction: It counts concordant pairs between the predicted risk score (e.g., prognostic index in AUTOSurv, log-risk function in CoxPH) and observed survival time^6,61^ and takes value between 0 and 1. C-index of 1 indicates perfect prediction and 0.5 is equivalent to random guessing.

5 $${\rm{C}}-{\rm{index}}=\frac{{\sum }_{i,j}{\bf{1}}\left\{{\eta }_{i} < {\eta }_{j}\right\}{\bf{1}}\{{T}_{i} > {T}_{j}\}{\delta }_{j}}{{\sum }_{i,j}{\bf{1}}\{{T}_{i} > {T}_{j}\}{\delta }_{j}}$$

Here ${\eta }_{i}$ and ${T}_{i}$ are the predicted risk score and overall follow-up time for patient $i$, respectively. The terms ${\boldsymbol{1}}\left\{\ldots \right\}$ and ${\delta }_{j}$ are both indicators: ${\boldsymbol{1}}\left\{\ldots \right\}$ takes value 1 if the argument in $\left\{\ldots \right\}$ is true and 0 otherwise; ${\delta }_{j}$ takes value 1 if the death of patient $j$ is observed and 0 if patient $j$ is censored.

### DeepSHAP for result interpretation

DeepSHAP is an activation-based interpretation approach. According to ref. ^25^, it avoids the saturation problem that perturbation- and gradient-based approaches fail to address. DeepSHAP shares the same key idea as DeepLIFT^25,26^. The core assumption of DeepLIFT is the summation-to-delta property:

6 $$\mathop{\sum }\limits_{k=1}^{{p}^{{\prime} }}{C}_{\Delta {x}_{k}\Delta t}=\Delta t$$

Here $t$ represents some output neuron of interest, and ${x}_{1},{x}_{2},\ldots ,{x}_{{p}^{{\prime} }}$ represent some neurons in the input layer or an intermediate layer that are necessary and sufficient to compute $t$. $\triangle t$ is the difference in output from some “reference” output, and $\triangle {x}_{k}$ is the difference in input from some “reference” input for ${x}_{k}$. ${C}_{\triangle {x}_{k}\triangle t}$ is the contribution score assigned to ${\triangle x}_{k}$ by DeepLIFT^25^. The summation-to-delta property hence states that the sum of the attributions over the input equals the difference-from-reference of the output^26^. Patients in the tuning set were assigned to the low-risk group if their predicted ${PI}$ were smaller than or equal to the median ${PI}$ (${{PI}}_{{med}}$), and patients were assigned to the high-risk group if their predicted ${PI}$ were higher than ${{PI}}_{{med}}$. During the interpretation procedures, we treated the low-risk group as the reference group. When backpropagating the predicted ${PI}$ values in the trained LFSurv model via DeepSHAP, we can compute SHAP values (the contribution scores in this setting, a SHAP value quantifies the average marginal impact of including an input across all conceivable orderings in which inputs can be included^25^) for the latent features and clinical variables and identify the features that contribute most to the difference in ${PI}$ between the low- and high-risk groups. Similarly, if we backpropagate the latent-feature values in the trained KL-PMVAE model via DeepSHAP, we can identify which genes/miRNAs/pathways contribute most to the difference in latent-feature values between the low- and high-risk groups. The +/− signs of the SHAP values imply the directions of the feature attributions, and higher absolute SHAP values correspond to greater contributions^24,26^. Following the procedure proposed by^26^, for each attempted DeepSHAP implementation, we randomly selected 100 samples from the low-risk group (which was our reference group) and 100 samples from the high-risk group, and for each feature its overall contribution was calculated by averaging its absolute SHAP values over the 100 high-risk group samples.

### Model training/tuning and KL-annealing

As mentioned earlier, for each dataset, 20% of the whole data were kept as testing set that did not participate in any of the model training/tuning process. The remaining 80% were denoted as tuning set and randomly split into 80% training and 20% validation sets (64% and 16% of the whole data, respectively). For each of the TCGA datasets at the developmental stage of AUTOSurv, random splitting of the tuning set was carried out 10 times, which gave us 10 different training/validation sets. For each of the splits, we trained the DNNs on the training set and conducted hyperparameter tuning using the validation set. The set of hyperparameters that gave the best model performance (i.e., lowest reconstruction loss for KL-PMVAE; or highest C-index for LFSurv) in the validation set were used to train the model on the whole tuning set. The trained model was then applied to the testing set to obtain the testing reconstruction loss/C-index. Ten different data splits yielded 10 testing C-indices, and their median, mean, and standard deviation (SD) were calculated and summarized in Supplementary Table 7. This scheme was applied on the same tuning set splits and the testing set data when tuning/testing other modeling strategies that we compared performance with. We summarized in Supplementary Table 4 the lists of hyperparameters that we tuned, and the strategies used to find the best sets of hyperparameters (e.g., number of nodes in hidden layer, learning rate, regularization parameter $\lambda$).

During framework evaluation, AUTOSurv was trained and tested on two non-TCGA datasets: ICGC-OVAU and Caldas-BC, which have smaller sample sizes (i.e., 93 subjects in the ICGC-OVAU dataset and 133 subjects in the Caldas-BC dataset) compared to the TCGA datasets. In order to mitigate the effect of randomness in data splitting and obtain more reliable results, for each of these two datasets the tuning/testing division (i.e., with an 80:20 ratio) was carried out 5 times. For each of the tuning/testing divisions, random splitting of the tuning set (i.e., into training/validation sets) was carried out 5 times. This gave us 25 different training/validation splits. Same as the procedure mentioned above, we trained AUTOSurv and other machine learning models on the training set and conducted hyperparameter tuning on the validation set. We then trained the models on the whole tuning set with the best sets of hyperparameters and reported the model performance on the testing set. Median, mean, and SD of the testing set C-indices are summarized in Supplementary Table 7.

During the external-cross-dataset validation, for each TCGA dataset, a pre-filtering process was conducted to exclude omics features whose min-max normalized expression data have low variance (<0.02) across all patients. The common clinical variables (age, clinical stage) and omics features between the pre-filtered TCGA dataset and its corresponding external independent validation dataset (i.e., between TCGA-OV and ICGC-OVAU; between TCGA-BRCA and Caldas-BC) were then selected for further analysis (see Supplementary Table 3 for a summary of the omics features). For each cancer type, we divided the TCGA dataset into internal training/validation sets with the ratio of 4:1 and repeated the data division process 10 times. For each division, we performed hyperparameter tuning via grid search and selected the set of hyperparameters that yielded the best performance on the internal validation set. We then trained the model on the entire TCGA dataset using the best hyperparameter set and tested its performance on the external validation dataset. Summary (median, mean, SD) of the highest C-index achieved by the models on the internal validation sets and their C-index on the external validation datasets (non-TCGA datasets) can be found in Supplementary Table 8.

We applied KL-annealing when training KL-PMVAE. During training, KL-annealing gradually increases $\beta$ value from 0 to 1 in the loss function (Eq. 3) and repeats this process for several cycles (the number of cycles and the cutting ratio in each cycle, illustrated in Supplementary Fig. 1, were also tuned as hyperparameters)^49^. When $\beta$ equals 0, the KL-divergence term has no influence on the loss function. The model learning is like a conventional autoencoder, which learns a point estimate for the latent variables. By gradually increasing $\beta$ to 1 at the first part of each cycle and placing more weight on the KL-divergence term, ${q}_{\phi }({\boldsymbol{z}}|{\boldsymbol{x}})$ is regularized to change from learning a point estimate to learning a distribution estimate. For the rest of each cycle, $\beta$ is fixed at value 1 to allow for optimizing the full VAE objective until convergence^49^. Because the learning process starts with random initialization, one key rationale behind KL-annealing is to prevent the distribution estimate from collapsing to the prior distribution (isotropic unit Gaussian $N({\boldsymbol{0}},{\boldsymbol{I}})$ in our case). In addition, according to the empirical results in ref. ^49^, KL-annealing has the potential to increase reconstruction ability for VAE. By applying KL-annealing we expect to increase the efficiency of KL-PMVAE in information extraction.

### Survival analysis

The survival outcomes of different predicted risk groups were presented using Kaplan–Meier (KM) curves. Cox proportional hazard (CoxPH) models were also used to study the association between the survival outcome and one or more variables. We used R (*survival*^62^, *survminer*^63^) and Python (*lifelines*^64^) packages to implement the survival analysis approaches.

### Statistical analysis

Two-sided Wilcoxon signed-rank test (non-parametric statistical hypothesis test for matched samples, since the C-indices are derived from the same set of patients) was applied for the pairwise comparison between testing set C-indices from different models or same models but in different cases (null hypothesis ${H}_{0}$: the median difference between the two sets of C-indices in comparison is equal to 0). A *p*-value $<$0.05 would suggest significant difference in prediction performance. Log-rank test was carried out to compare KM curves (null hypothesis ${H}_{0}$: there is no difference in the probability of an event at any time point between groups), A *p*-value < 0.05 would suggest significant difference in survival outcomes between the two groups in comparison. For the univariate CoxPH analysis regarding the identified key genes, hazard ratio (HR) with 95% confidence interval (CI) for each gene and likelihood ratio test (null hypothesis ${H}_{0}$: there is no significant association between the regressor and the survival outcome) *p*-value were reported, and multiple testing was adjusted using Benjamini–Hochberg (BH) method.

### Reporting Summary

Further information on research design is available in the Nature Portfolio Reporting Summary linked to this article.

### Supplementary information

Supplementary Materials

Reporting Summary Checklist

## Data Availability

Overall survival time and censoring status, demographic/clinical information, and omics data of breast cancer and ovarian cancer patients were obtained from UCSC Xena data portal (https://xenabrowser.net/datapages/) and International Cancer Genome Consortium (ICGC) data portal (https://dcc.icgc.org/) for the Genomic Data Commons (GDC) BRCA cohort (https://xenabrowser.net/datapages/?cohort=GDC%20TCGA%20Breast%20Cancer%20 (BRCA)&removeHub=https%3A%2F%2Fxena.treehouse.gi.ucsc.edu%3A443) and OV cohort (https://xenabrowser.net/datapages/?cohort=GDC%20TCGA%20Ovarian%20Cancer%20 (OV)&removeHub=https%3A%2F%2Fxena.treehouse.gi.ucsc.edu%3A443) of The Cancer Genome Atlas (TCGA) program, the Caldas 2007 Breast cancer cohort (https://xenabrowser.net/datapages/?cohort=Breast%20Cancer%20 (Caldas%202007)&removeHub=https%3A%2F%2Fxena.treehouse.gi.ucsc.edu%3A443), and the ICGC Ovarian Cancer – Australian (OVAU) cohort (https://dcc.icgc.org/releases/current/Projects/OV-AU). The Reactome pathway information was obtained from the online resource Database for Annotation, Visualization, and Integrated Discovery (DAVID) (https://david.ncifcrf.gov/home.jsp). R codes of data preprocessing procedures were released on our Github website (https://github.com/jianglindong93/AUTOSurv) with some preprocessed data examples. More details on data collection including URL links to specific data types can also be found on our Github website.

## Code availability

We released AUTOSurv with source code, executable file, testing data, and tutorials at https://github.com/jianglindong93/AUTOSurv.
