# Supplementary material for: Autosurv: interpretable deep learning framework for cancer survival analysis incorporating clinical and multi-omics data
Source: NPJ Precis Oncol. 2024 Jan 5;8:4. doi: 10.1038/s41698-023-00494-6 (PMC10770412; doi:10.1038/s41698-023-00494-6)
Supplement: Supplementary file 1 — Supplementary Materials [file 41698_2023_494_MOESM1_ESM.pdf]

Supplementary materials

Table of contents:

1. Summarization of demographic/clinical information … … … (3-4)

Supplementary Table 1

2. Information regarding omics data … … … (5-6)

Generation pipelines

Chromosome information

Omics data summarization: Supplementary Table 2-3

3. Hyperparameter tuning … … … (7-8)

Hyperparameters for AUTOSurv: Supplementary Table 4.1-2

Hyperparameters for the machine learning approaches: Supplementary Table 4.3

4. Illustration for KL-annealing … … … (9)

Supplementary Figure 1

5. Alterations of AUTOSurv … … … (10)

Supplementary Figure 2

6. Additional performance comparisons … … … (11-13)

Supplementary Figure 3

Supplementary Figure 4

7.Additional Kaplan Meier curves … … … (14-19)

Supplementary Figure 5

8. Partial supports found in previous literatures for interpretation results … … … (20-21)

Supplementary Table 5

9. Identified key input factors and key pathway factors … … … (22-23)

Supplementary Figure 6

Supplementary Figure 7

10. Univariate CoxPH analysis for identified key genes … … … (24)

Supplementary Table 6

11. Summarization of model performance … … … (25-28)

Supplementary Table 7

Supplementary Table 8

References … … … (29-30)

1. Summarization of demographic/clinical information

Supplementary Table 1: Demographic and clinical characteristics of patients from different datasets

TCGA-BRCA (Total sample size 1,058, death count = 175 [16.541%])			
	Characteristics	Descriptive statistics	*P-value (Overall survival)
	Age at diagnosis, mean ± SD	57.48 ± 13.03	< 0.0001
	Race, N (%)		
	American Indian or Alaska Resident or Asian	59 (5.58%)	0.398
	Black or African American	178 (16.82%)	
	White	821 (77.60%)	
	Disease stage, N (%)		
	Stage I	193 (18.24%)	<0.0001
	Stage II	608 (57.47%)	
	Stage III	240 (22.68%)	
	Stage IV	17 (1.61%)	
TCGA-OV (Total sample size 355, death count = 222 [62.535%])			
	Characteristics	Descriptive statistics	*P-value (Overall survival)
	Age at diagnosis, mean ± SD	59.61 ± 11.29	0.0003
	Race, N (%)		
	American Indian or Alaska native or Asian or native Hawaiian or other Pacific Islander	15 (4.23%)	0.018
	Black or African American	24 (6.76%)	
	White	316 (89.01%)	
	Disease stage, N (%)		
	Stage I	1 (0.28%)	0.04
	Stage II	22 (6.2%)	
	Stage III	278 (78.31%)	
	Stage IV	54 (15.21%)	
	Histological grade, N (%)		
	G1	1 (0.28%)	0.429
	G2	44 (12.39%)	
	GB	2 (0.56%)	
	G3	307 (86.48%)	
	G4	1 (0.28%)	
Caldas-BC (Total sample size 133, death count = 35 [26.316%])			
	Characteristics	Descriptive statistics	*P-value (Overall survival)
	Age at diagnosis, mean ± SD	56.95 ± 8.92	0.076

	Tumor size in cm, mean ± SD	1.86 ± 0.70	0.004
	Menopausal status, N (%)		
	Premenopausal	43 (32.33%)	0.683
	Postmenopausal	90 (67.67%)	
	Disease stage, N (%)		
	Stage I	90 (67.67%)	0.011
	Stage II	32 (24.06%)	
	Stage III	11 (8.27%)	
	Histological grade, N (%)		
	G1	35 (26.32%)	0.001
	G2	50 (37.59%)	
	G3	48 (36.09%)	
ICGC-OVAU (Total sample size 93, death count = 74 [79.57%])			
	Characteristics	Descriptive statistics	*P-value (Overall survival)
	Age at diagnosis, mean ± SD	59.63 ± 8.67	0.5
	Treatment (surgery [and chemotherapy]), N (%)		
	Yes	45 (48.39%)	0.235
	No	48 (51.61%)	
	Disease stage, N (%)		
	Stage III	79 (84.95%)	0.129
	Stage IV	14 (15.05%)	
	Diagnosis iCD10, N (%)		
	C48.2 (Malignant neoplasm of peritoneum, unspecified)	18 (19.35%)	0.033
	C56 (Malignant neoplasm of ovary)	73 (78.49%)	
	C57 (Malignant neoplasm of other and unspecified female genital organs)	2 (2.15%)	

**For each dataset, preliminary analysis was conducted by fitting CoxPH model using each of the variables respectively. P-values computed via likelihood ratio tests. TCGA-BRCA: the Genomic Data Commons (GDC) Breast Cancer (BRCA) cohort of The Cancer Genome Atlas (TCGA) program; during model fitting, patients were categorized into "white" and "other races"; "stage I", "stage II", and "stage III or higher". TCGA-OV: the Genomic Data Commons (GDC) Ovarian Cancer (OV) cohort of The Cancer Genome Atlas (TCGA) program; during model fitting, patients were categorized into "white" and "other races"; "stage III or higher" and "lower stages"; "G3 or higher" and "lower grades". Caldas-BC: the UCSC Xena Caldas 2007 Breast Cancer cohort; during model fitting, patients were categorized into "premenopausal" and "postmenopausal"; "stage I" and "higher stages"; "G1", "G2" and "G3". ICGC-OVAU: International Cancer Genome Consortium (ICGC) Ovarian Cancer – Australian (OVAU) cohort; during model fitting, patients were categorized into "treated" and "untreated"; "stage III" and "stage IV"; "C56", and "other iCD10 diagnosis".*

2. Information regarding omics data

Generation pipelines

Detailed information about the GDC pipelines applied to generate the gene and miRNA expression data on UCSC Xena portal can be found on the following websites:

TCGA data:

Gene expression RNAseq	https://docs.gdc.cancer.gov/Data/Bioinformatics_Pipelines/Expression_mRNA_Pipeline/
MiRNA expression quantification	https://docs.gdc.cancer.gov/Data/Bioinformatics_Pipelines/miRNA_Pipeline/

For the Caldas-BC cohort, information about the pipeline applied to generate the gene expression data can be found on [1].

Chromosome information

For TCGA-BRCA, TCGA-OV, and ICGC-OVAU datasets, we obtained chromosome information for the genes using R package *biomaRt*. UCSC Xena and ICGC portal provide Ensemble IDs for the genes. For Caldas-BC cohort, Xena provides Agilent ID for the genes and chromosome information for the genes were obtained from DAVID. During preprocessing, a gene was removed if its ID cannot get a matched chromosome.

Omics data summarization

Supplementary Table 2: Summary of the gene/miRNA expression data

Dataset	Number of features before filtering process and adaptation for pathway-masking design: genes / miRNAs	Number of features after filtering process and adaptation for pathway-masking design: genes / miRNAs
TCGA-BRCA	60,483 / 1,881	2,699 / 516
TCGA-OV	60,483 / 1,881	5,147 / 865
Caldas-BC	17,080 / --	~
ICGC-OVAU	56,206 / 800	~

For the filtering process, we first filtered out genes that are not on the autosomes or X chromosome, then the original expression data were min-max normalized across all patients in the tuning set and genes/miRNAs with variance of < 0.02 were excluded. Adaptation for pathway-masking design: *Genes that do not belong to any of the Reactome pathways were excluded. TCGA-BRCA: GDC TCGA Breast Cancer cohort; TCGA-OV: GDC TCGA Ovarian Cancer cohort; Caldas-BC: UCSC Xena Caldas 2007 Breast Cancer cohort; ICGC-OVAU: International Cancer Genome Consortium Ovarian Cancer Australian cohort.*

For ***Caldas-BC*** *and* ***ICGC-OVAU*** *datasets, number of features after filtering process and adaptation for pathway-masking design varies for different* ***tuning/testing*** *set divisions:*

Dataset	Number of features: genes/miRNAs				
	Division 1	Division 2	Division 3	Division 4	Division 5
Caldas-BC	4,197/--	3,998/--	4,196/--	3,958/--	4,189/--
ICGC-OVAU	8,362/198	8,470/721	8,342/196	8,471/714	8,739/753

For BRCA and OV, number of omics features before and after pre-filtering (on TCGA datasets) and matching between TCGA and non-TCGA datasets during external-cross-dataset validation:

Supplementary Table 3: Summary of the gene/miRNA expression data during external-cross-dataset validation

Cancer type	*Dataset*	*Number of features before pre-filtering and TCGA/non-TCGA matching: genes/miRNAS*	*Number of features after pre-filtering and TCGA/non-TCGA matching: genes/miRNAS*
BRCA	*TCGA-BRCA*	*60,483 / --*	*1367/--*
	Caldas-BC	*17,080 / --*	
OV	*TCGA-OV*	*60,483 / 1,881*	*4783/396*
	ICGC-OVAU	*56,206 / 800*	

Note: the genes are also subject to adaptation for pathway-masking design as mentioned above.

3. Hyperparameter tuning

Hyperparameters for AUTOSurv

The best set of hyperparameters were found via grid search.

Supplementary Table 4.1 – Hyperparameters for KL-PMVAE

Hyperparameter	***Values tuned***
Number of latent features in bottleneck layer	*8, 16, 32, 64*
Learning rate	*0.1, 0.05, 0.005, 0.001*
Weight decay (i.e., regularization parameter λ_1)	*0.1, 0.05, 0.005, 0.0005*
Number of epochs	*800, 1200, 1600, 2000, 2400*
Number of cycles	*2, 4, 5, 10*
Cutting ratio	*0.3, 0.5, 0.7, 0.9*

The set of hyperparameters that yielded the lowest reconstruction loss were selected as the best set of hyperparameters.

Supplementary Table 4.2 – Hyperparameters for LFSurv

Hyperparameter	***Values tuned***
Number of nodes in the hidden layer	*4, 8, 16, 32*
Learning rate	*0.3, 0.1, 0.05, 0.01, 0.0075*
Weight decay (i.e., regularization parameter λ_2)	*0.001, 0.00075, 0.0005, 0.00025, 0.0001*
Dropout rate for input layer	*0.1, 0.3, 0.5*
Dropout rate for hidden layer	*0.1, 0.3, 0.5*

The set of hyperparameters that yielded the highest C-index were selected as the best set of hyperparameters.

Hyperparameters for the machine learning approaches

- Cox proportional hazard model with elastic net regularization (CoxPH-ENet) model was implemented using *CoxPHFitter* function in Python package *lifelines*.
- Random survival forest (RSF) model was implemented using *RandomSurvivalForest* function in Python package *sksurv*.
- Extreme gradient boosting (XGBoost) with CoxPH (XGB-CoxPH) and XGBoost with accelerated failure time (XGB-AFT) were implemented using *xgb* function in Python package *xgboost*.

Supplementary Table 4.3 – Hyperparameters for machine learning approaches

Model name	***Hyperparameter***	***Values tuned***	***Tuning strategy***
CoxPH-ENet	*α*	*0, 0.01, 0.05, 0.1, 0.5, 1, 5*	*Grid search*
	L1 ratio	*0, 0.3, 0.5, 0.7, 0.9, 1*	
RSF	*n_estimators*	*100, 200, 500, 1000*	*Grid search*
	min_samples_split	*4, 6, 8, 10, 12*	
	min_samples_leaf	*3, 6, 9, 12, 15*	
XGB-CoxPH	*eta*	*0.002*	*Optuna* [2]
	objective	*survival:cox*	
	eval_metric	*cox-nloglik*	
	subsample	*0.5*	

	learning_rate	*Range from 0.0001 to 0.01*	
	lambda	*Range from 1e-8 to 1.0*	
	alpha	*Range from 1e-8 to 1.0*	
	max_depth	*Range from 3 to 8*	
XGB-AFT	*objective*	*survival:aft*	*Optuna*
	eval_metric	*aft-nloglik*	
	tree_method	*hist*	
	learning_rate	*Range from 0.001 to 0.1*	
	aft_loss_distribution	*'normal', 'logistic', 'extreme'*	
	aft_loss_distribution_scale	*Range from 0.1 to 10*	
	lambda	*Range from 1e-8 to 1.0*	
	alpha	*Range from 1e-8 to 1.0*	
	max_depth	*Range from 3 to 8*	

4. Illustration for KL-annealing

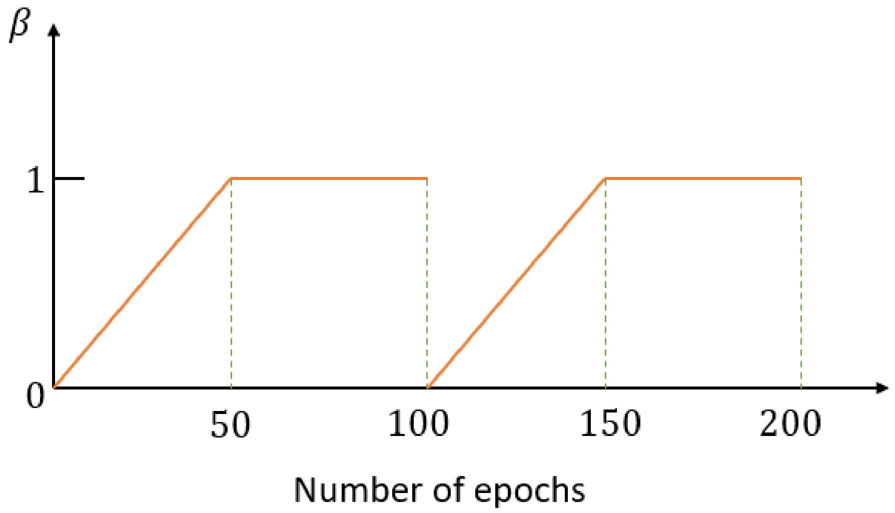

Supplementary Figure 1: Example of KL-annealing learning scheme. In this example the total number of epochs is 200, there are 2 cycles, each with 100 epochs, and the cutting ratio within each cycle is 50:50. In the first part of each cycle (i.e., 50 epochs), the value of β gradually increases from 0 to 1, and in the second part of the cycle the value of β is fixed at 1. β value will change to 0 at the beginning of the next cycle and the process repeats.

5. Alterations of AUTOSurv

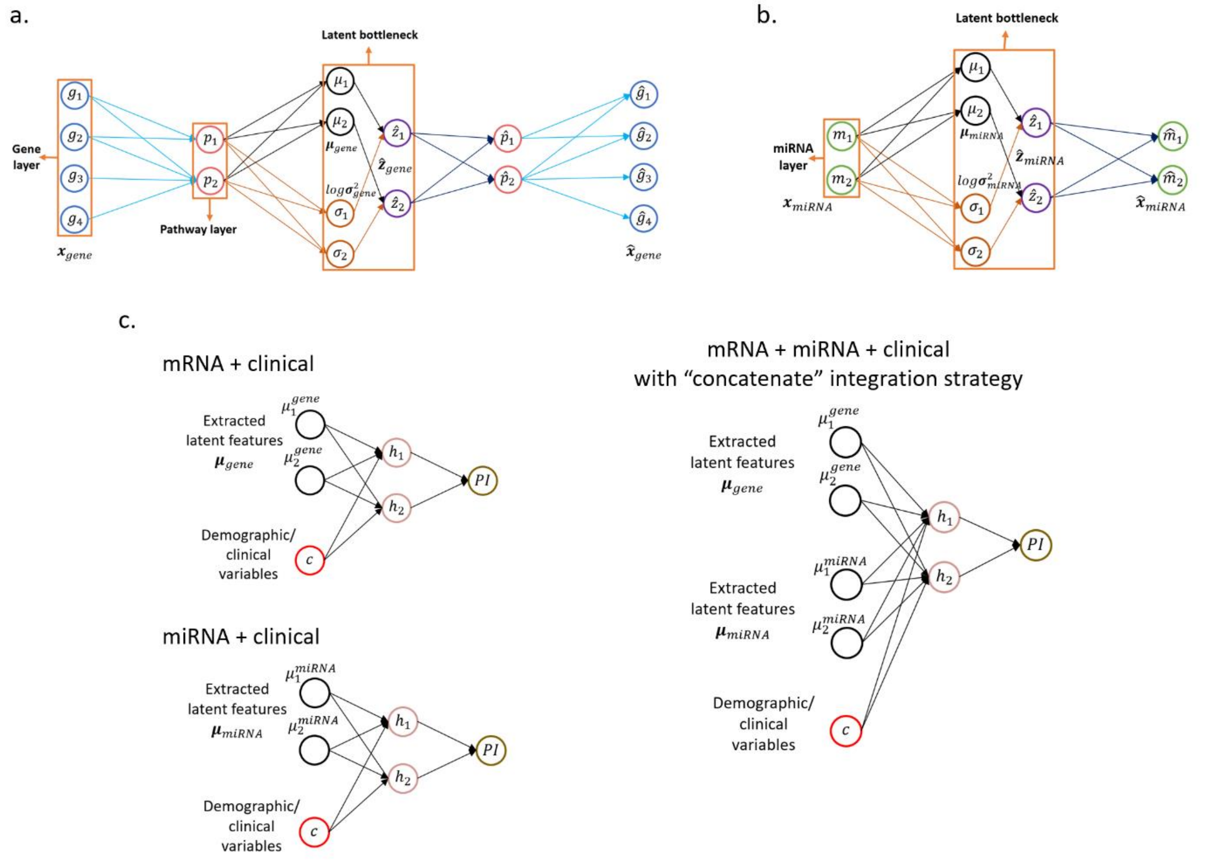

Supplementary Figure 2: Alterations of AUTOSurv. (a) Modified KL-PMVAE to extract latent features $\boldsymbol{\mu}_{gene}$ *from gene expression data. (b) Modified KL-PMVAE to extract latent features* $\boldsymbol{\mu}_{miRNA}$ *from miRNA expression data. (c) LFSurv network taking different input data: in the "mRNA + clinical" case, latent features* $\boldsymbol{\mu}_{gene}$ *and demographic/clinical variables (e.g., age, disease stage, race) were taken as input; in the "miRNA + clinical" case, latent features* $\boldsymbol{\mu}_{miRNA}$ *and demographic/clinical variables were taken as input; in the "mRNA + miRNA + clinical" case with "concatenate" integration strategy, latent features* $\boldsymbol{\mu}_{gene}$ *and* $\boldsymbol{\mu}_{miRNA}$ *were concatenated directly and the concatenated features together with demographic/clinical variables were fed into LFSurv as input.*

6. Additional performance comparisons

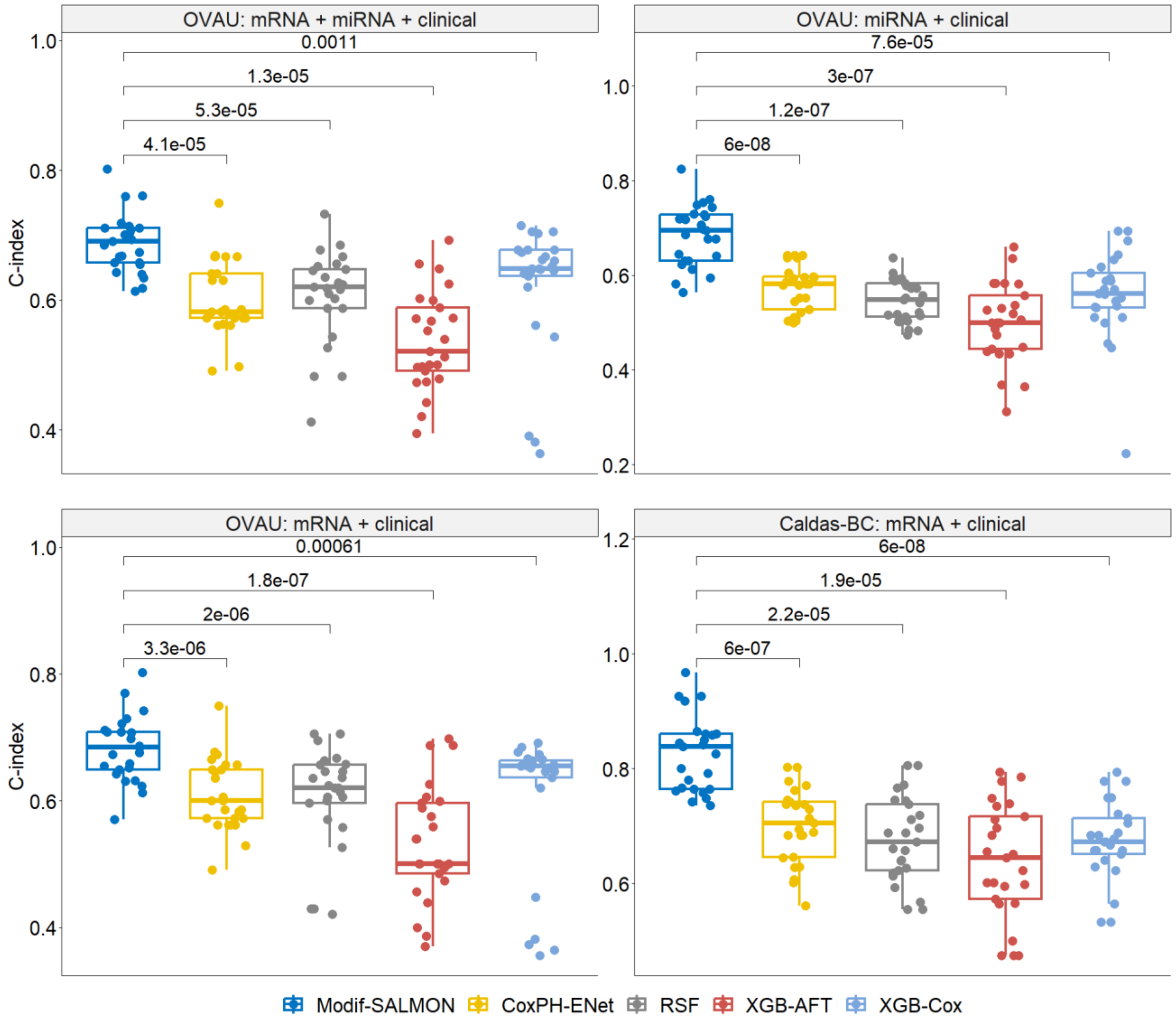

Supplementary Figure 3 – Performance comparison between Modified-SALMON and the machine learning methods in two external validation datasets: ICGC-OVAU and Caldas-BC. Top-left: the "mRNA + miRNA + clinical" case in ICGC-OVAU dataset; top-right: the "miRNA + clinical" case in ICGC-OVAU dataset; lower-left: the "mRNA + clinical" case in ICGC-OVAU dataset; lower-right: the "mRNA + clinical" case in Caldas-BCt. Note that Caldas-BC does not have miRNA expression data. CoxPH-ENet: Cox Proportional Hazard model with Elastic Net; RSF: Random Survival Forest; XGB-AFT: Extreme Gradient Boosting with Accelerated Failure Time; XGB-Cox: Extreme Gradient Boosting with CoxPH. The p-value from two-sided Wilcoxon signed rank test (i.e., null hypothesis H_0: median difference is equal to 0; versus alternative hypothesis H_A: median difference is not 0) is displayed between boxes.

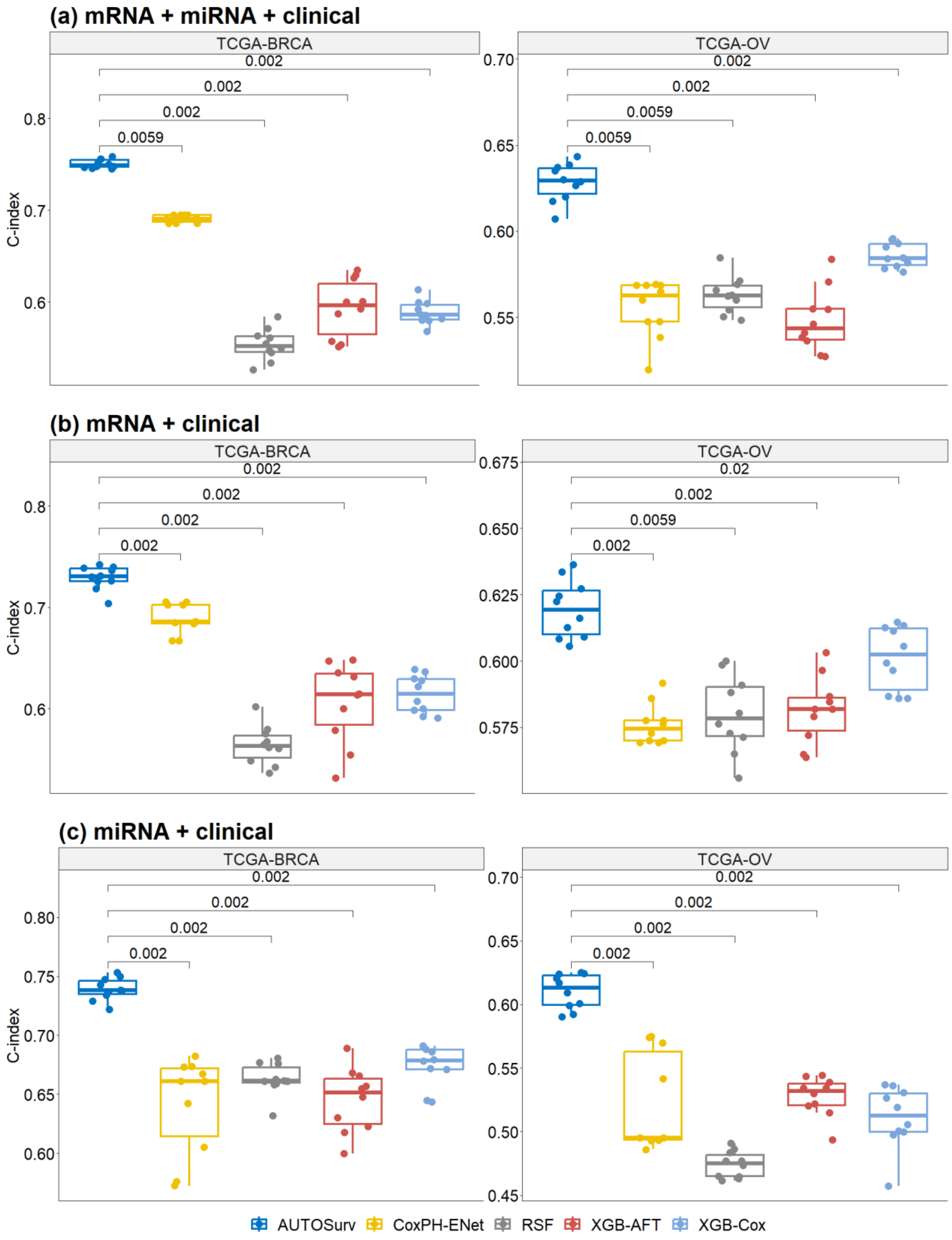

Supplementary Figure 4 – Performance comparison between AUTOSurv and the machine learning methods in the TCGA datasets: TCGA-BRCA (left column) and TCGA-OV (right column). CoxPH-ENet: Cox Proportional

Hazard model with Elastic Net; RSF: Random Survival Forest; XGB-AFT: Extreme Gradient Boosting with Accelerated Failure Time; XGB-Cox: Extreme Gradient Boosting with CoxPH. The p-value from two-sided Wilcoxon signed rank test (i.e., null hypothesis H_0: median difference is equal to 0; versus alternative hypothesis H_A: median difference is not 0) is displayed between boxes.

7. Additional Kaplan Meier curves

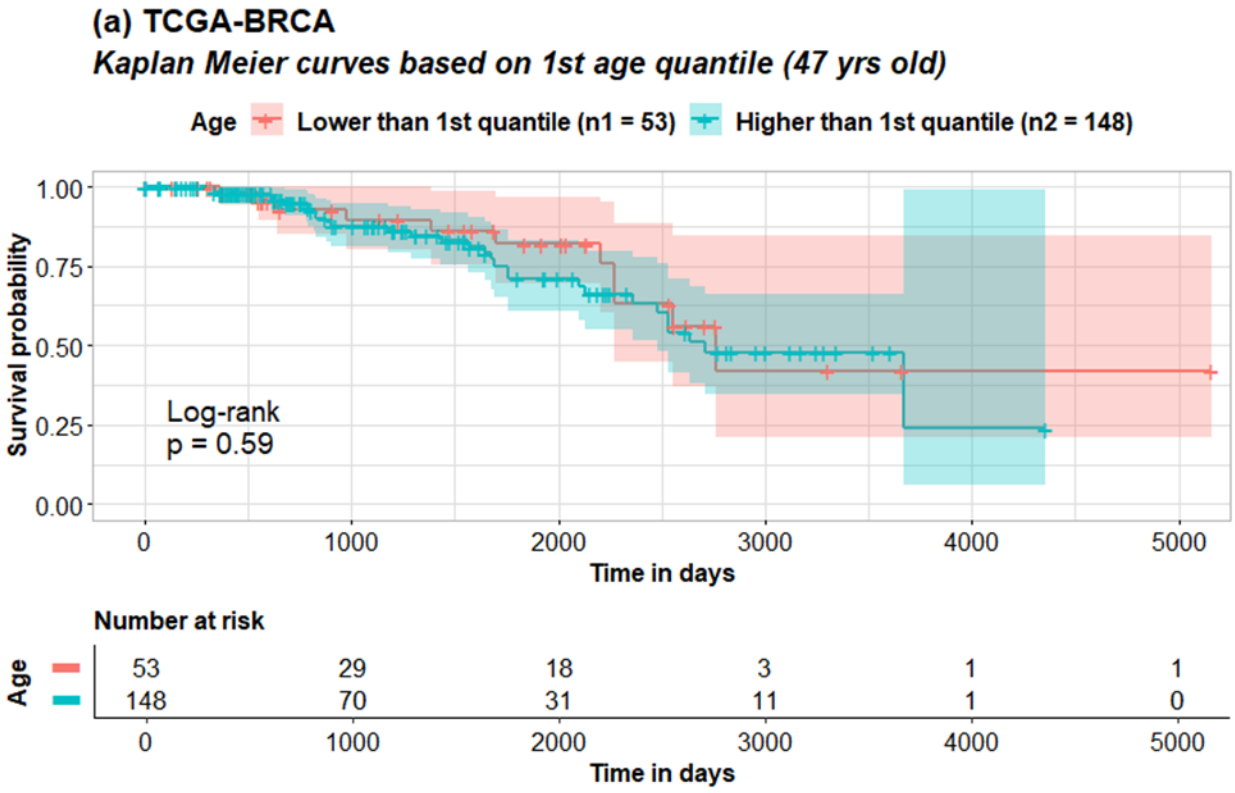

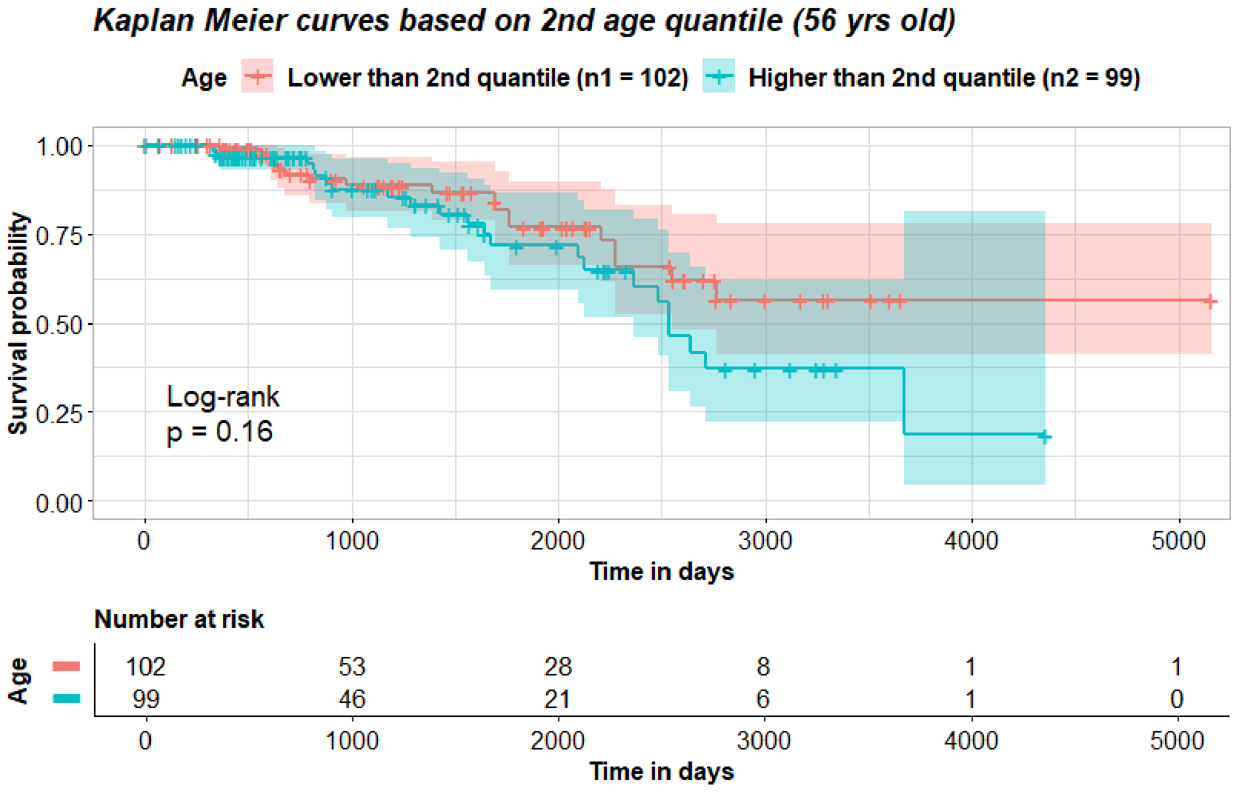

Kaplan Meier curves based on 3rd age quantile (68 yrs old)

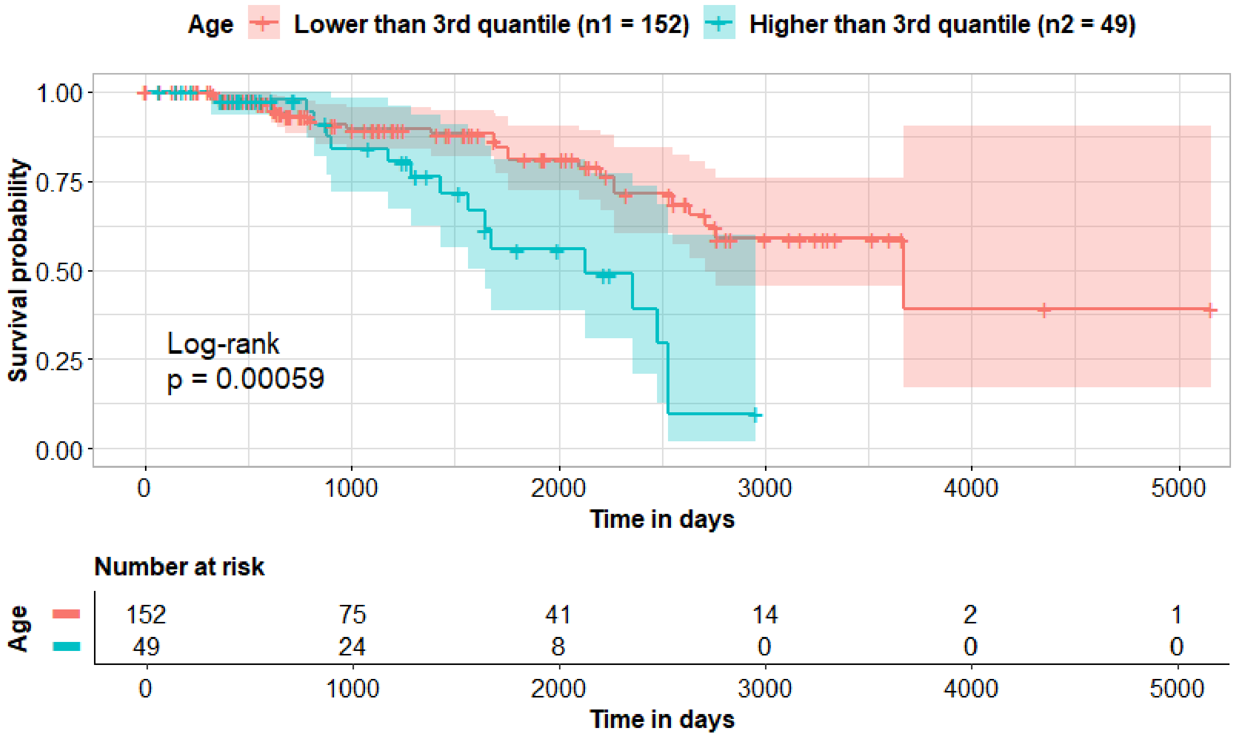

Kaplan Meier curves based on disease stage

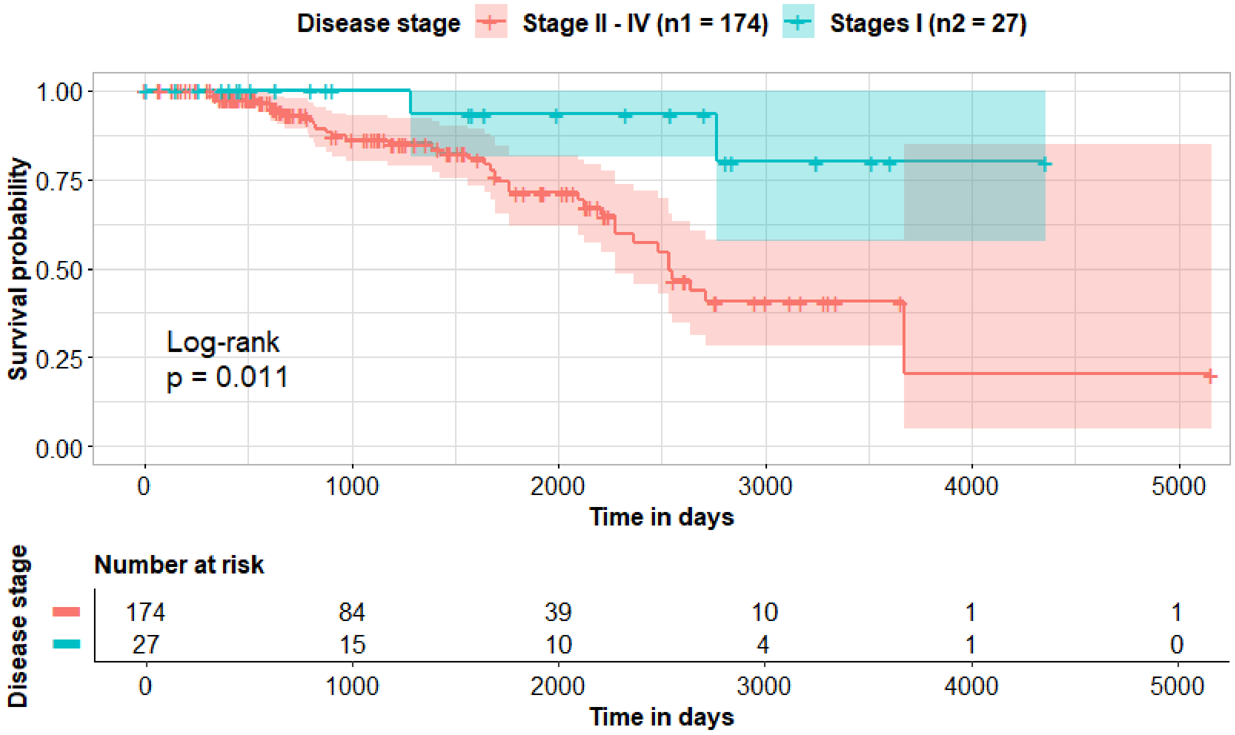

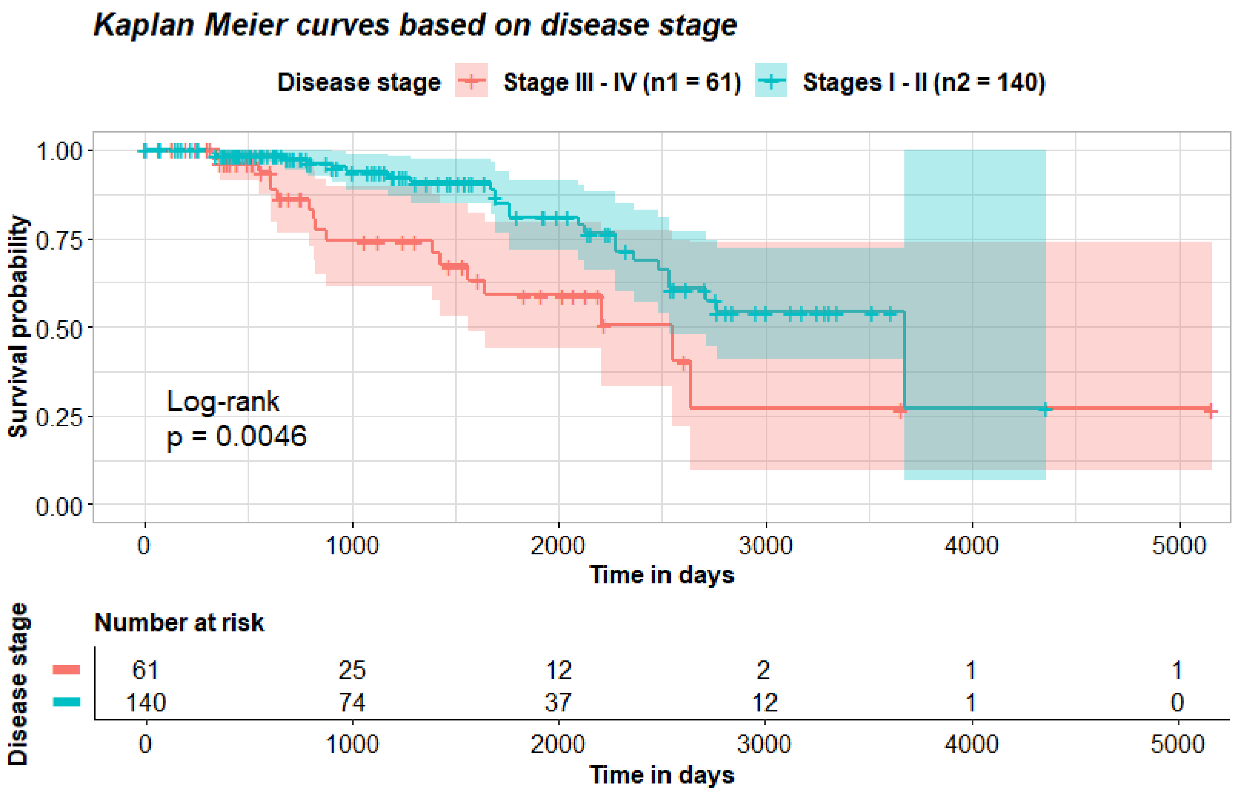
Kaplan Meier curves based on disease stage
Disease stage
Stage III - IV (n1 = 61)
Stages I - II (n2 = 140)
Survival probability
1.00
0.75
0.50
0.25
0.00
Log-rank
p = 0.0046
0
1000
2000
3000
4000
5000
Time in days
Number at risk
Disease stage
61 25 12 2 1 1
140 74 37 12 1 0
0
1000
2000
3000
4000
5000
Time in days

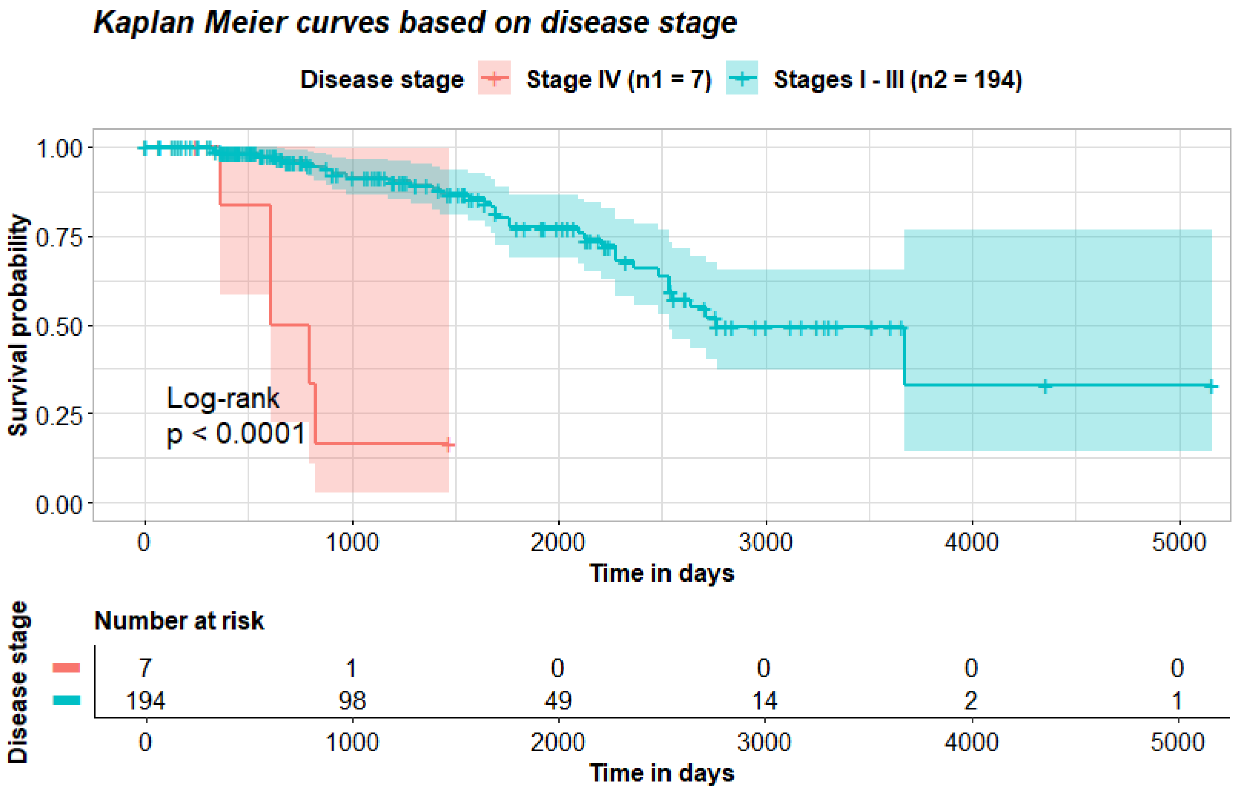
Kaplan Meier curves based on disease stage
Disease stage
Stage IV (n1 = 7)
Stages I - III (n2 = 194)
Survival probability
1.00
0.75
0.50
0.25
0.00
Log-rank
p < 0.0001
0
1000
2000
3000
4000
5000
Time in days
Number at risk
Disease stage
7 1 0 0 0 0
194 98 49 14 2 1
0
1000
2000
3000
4000
5000
Time in days

(b) TCGA-OV

Kaplan Meier curves based on 1st age quantile (50 yrs old)

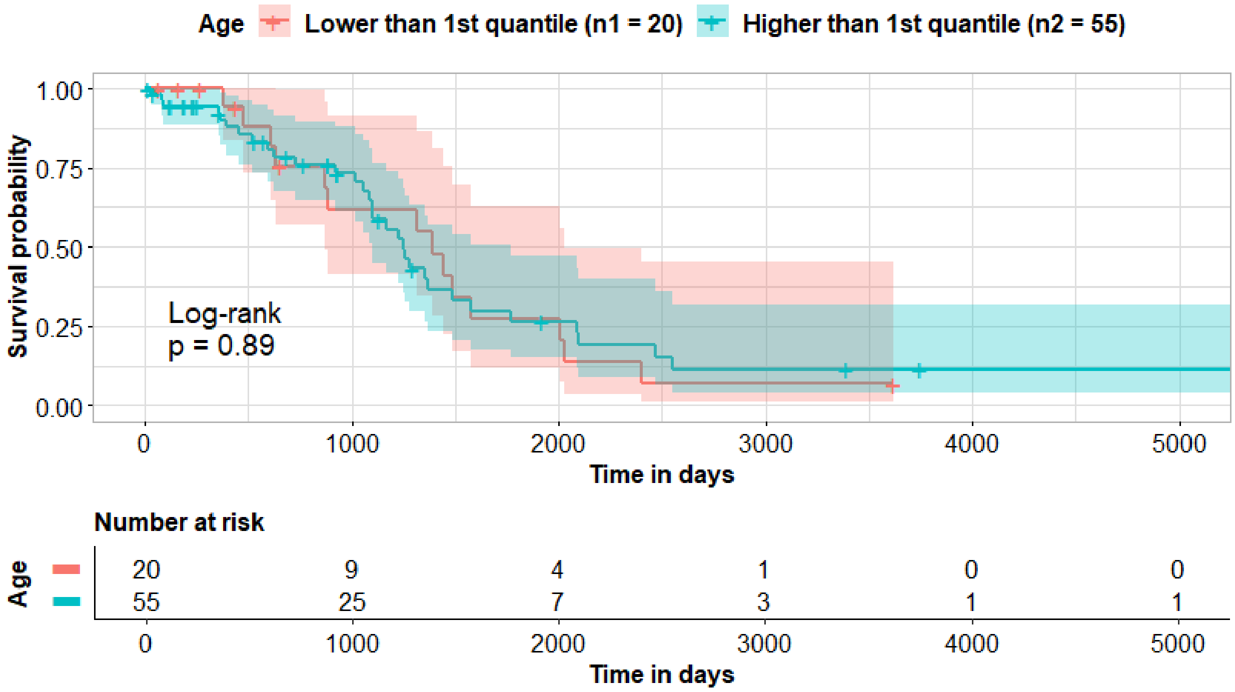

Kaplan Meier curves based on 2nd age quantile (59 yrs old)

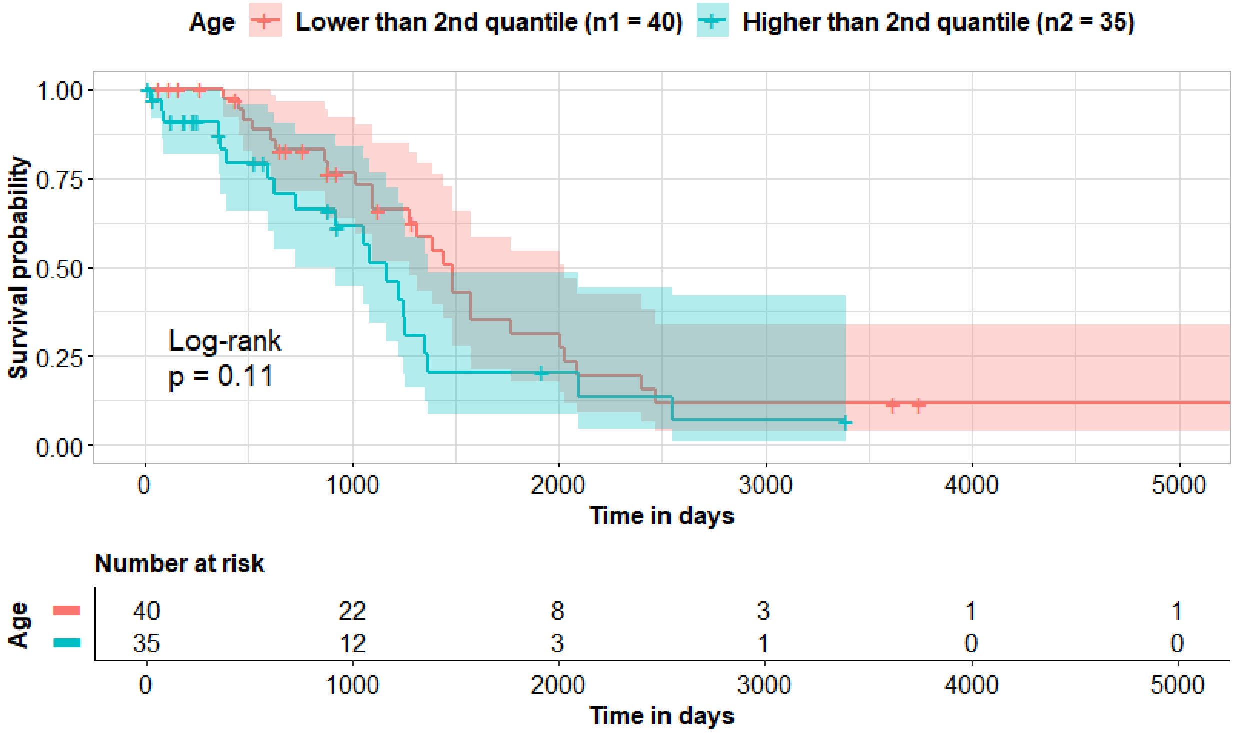

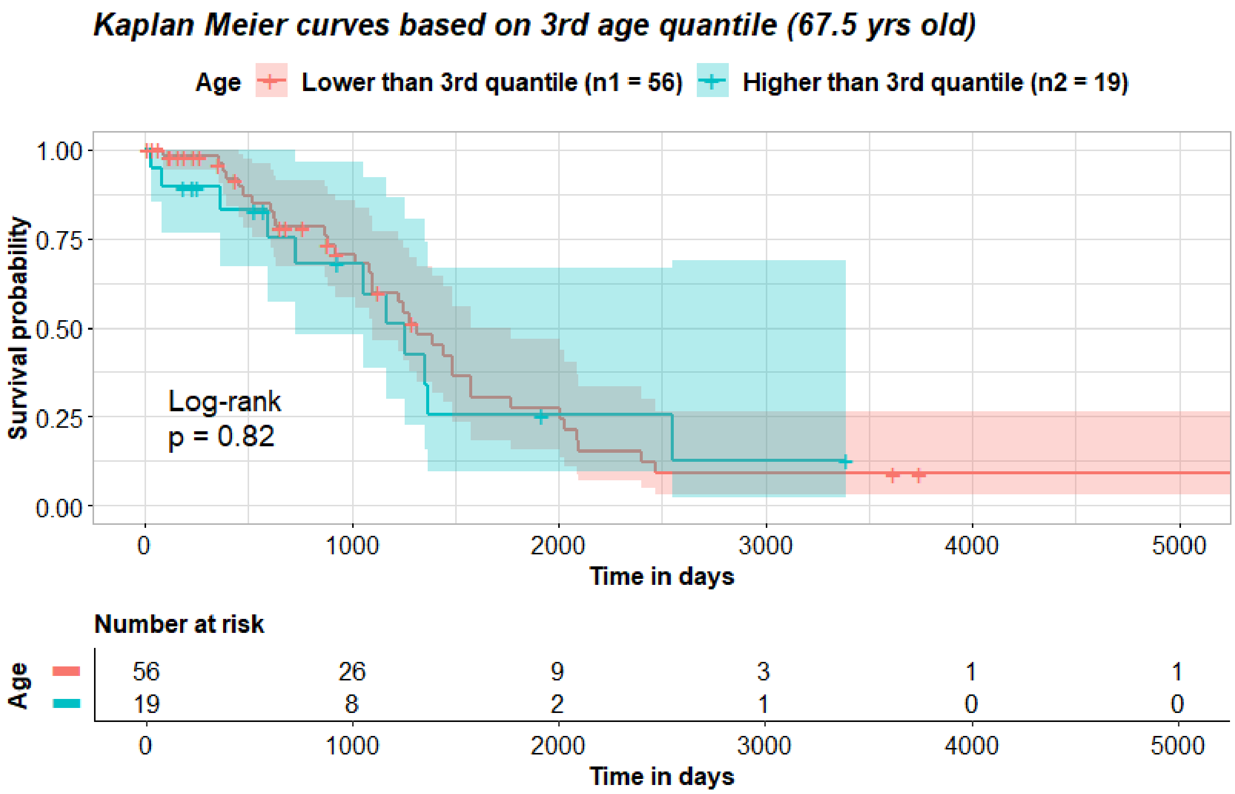

Kaplan Meier curves based on 3rd age quantile (67.5 yrs old)
Age
Lower than 3rd quantile (n1 = 56)
Higher than 3rd quantile (n2 = 19)
Survival probability
1.00
0.75
0.50
0.25
0.00
Log-rank
p = 0.82
0
1000
2000
3000
4000
5000
Time in days
Number at risk
Age
56
26
9
3
1
1
19
8
2
1
0
0
0
1000
2000
3000
4000
5000
Time in days

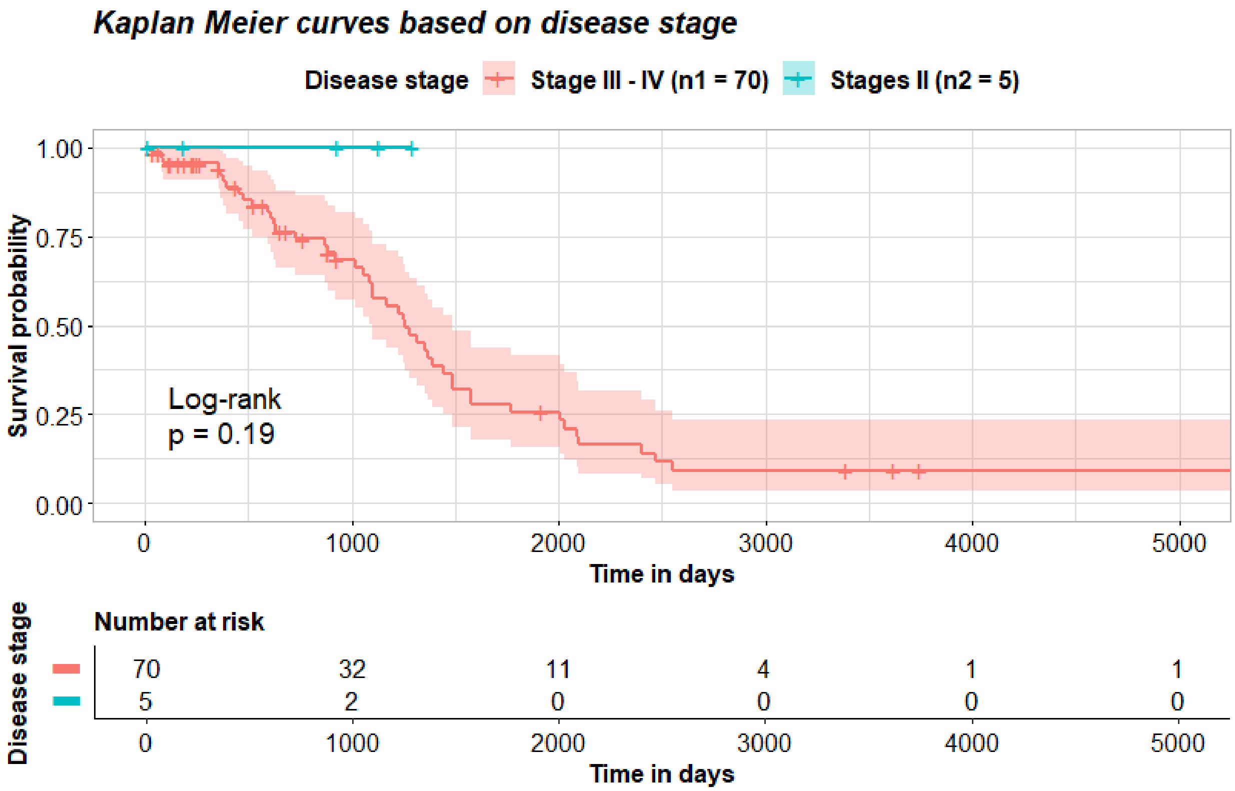

Kaplan Meier curves based on disease stage
Disease stage
Stage III - IV (n1 = 70)
Stages II (n2 = 5)
Survival probability
1.00
0.75
0.50
0.25
0.00
Log-rank
p = 0.19
0
1000
2000
3000
4000
5000
Time in days
Number at risk
Disease stage
70
32
11
4
1
1
5
2
0
0
0
0
0
1000
2000
3000
4000
5000
Time in days

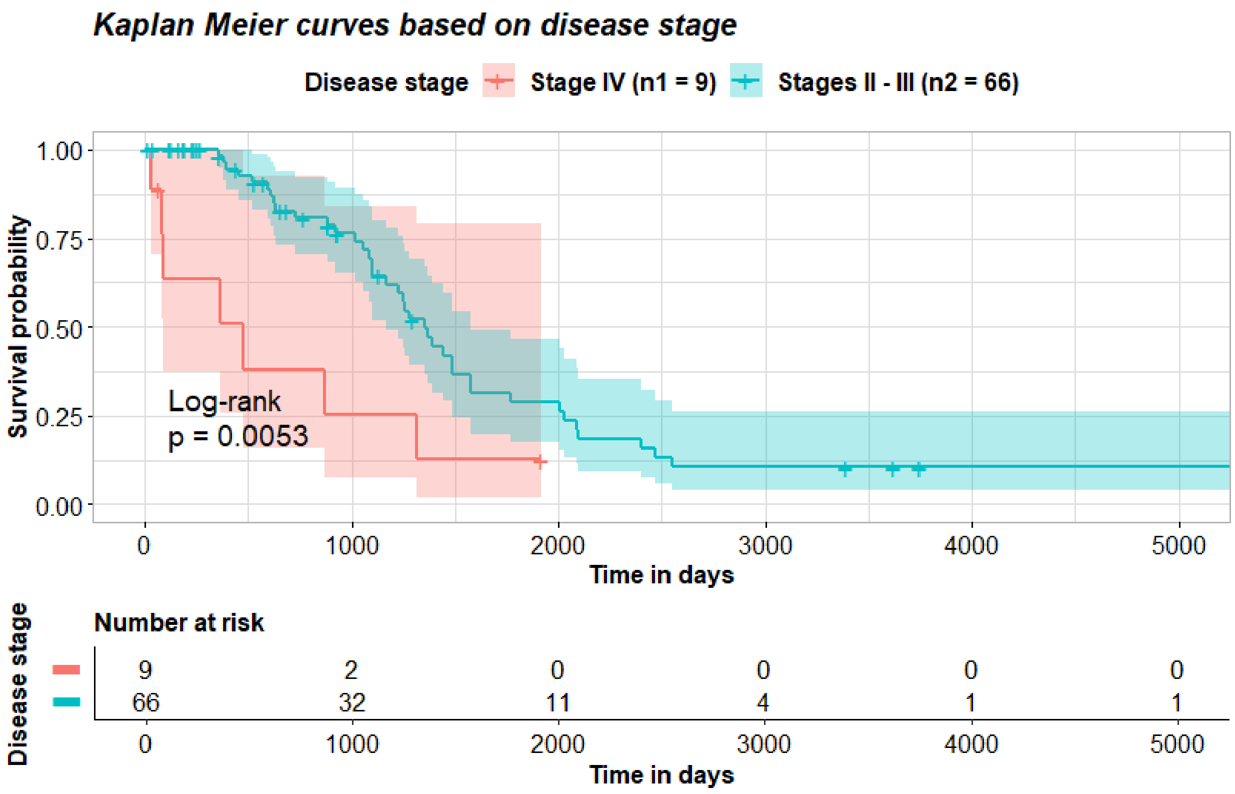

Supplementary Figure 5 – Kaplan Meier curves based on age and disease stage for testing set patients in (a) TCGA-BRCA dataset; and (b) TCGA-OV dataset.

8. Partial supports found in previous literatures for interpretation results

Supplementary Table 5 – Summarization and reference list of supporting evidence for DeepSHAP interpretation results on TCGA datasets

**TCGA-BRCA**		
**Key input factors**	**Evidence of potential association with breast cancer**	**Reference**
CD3G	Expression of CD3G in primary tumors of the breast was found to be correlated with overall survival of lymph node negative patients.	[3]
CDC20	1. High CDC20 immuno-expression alone/in combination with high securin immuno-expression indicated significantly higher risk of death from breast cancer.<br>2. CDC20 knockdown inhibited the migration of metastatic MDA-MB-231 breast cancer cell line.	[4,5]
FABP4	FABP4 promotes obesity-associated breast cancer development.	[6]
FGF2	FGF2 expression was revealed to be correlated with the presence of cancer-associated fibroblasts (CAFs), and a more proliferated phenotype was manifested in CAF-conditioned-media-treated human breast cancer MDA-MB-231 cells.	[7]
PIK3R1	It was suggested that in breast cancer, the suppression of cell growth, migration, and invasion is achieved, in part, through the inhibition of PI3K/AKT activation via direct targeting of PIK3R1, as well as the reversal of EMT, by means of miR-21 knockdown.	[8]
PLCG2	PLCG2, identified as an irradiation-responsive gene, holds the potential of being a modifier of breast cancer risk in individuals carrying BRCA2 mutations.	[9,10]
PLIN1	PLIN1's mRNA expression is significantly downregulated in human breast cancer.	[11]
PSMB9	PSMB9 was found to be overexpressed in breast cancer cells.	[12]
RFC4	RFC4 was a predictor of poorer overall survival in breast cancer.	[13,14]
TFAP2B	Knockdown of TFAP2B resulted in reduced proliferation of lobular breast cancer cell lines in vitro, highlighting TFAP2B's role in controlling tumor cell proliferation within the slow-growing subtype of breast cancer.	[15,16]
TLR4	TLR4 plays an important role in either eliminating breast cancer cells or inducing breast cancer development, as well as facilitating the transformation of normal cells.	[17]
**Key pathway factors**	**Pathway names**	**References***
R-HSA-72695	Formation of the ternary complex, and subsequently, the 43S complex.	[18,19]
R-HSA-163560	Triglyceride catabolism.	[20,21]
R-HSA-202430	Translocation of ZAP-70 to Immunological synapse.	[22]
R-HSA-418555	G alpha (s) signaling events.	[23]
R-HSA-909733	Interferon alpha/beta signaling.	[24-27]

R-HSA-975957	Nonsense Mediated Decay (NMD) enhanced by the Exon Junction Complex (EJC).	[28,29]
R-HSA-3000178	ECM proteoglycans.	[30]
R-HSA-3232118	SUMOylation of transcription factors.	[31]
R-HSA-5656169	Termination of translesion DNA synthesis.	[32]
**TCGA-OV**		
**Key input factors**	**Evidence of potential association with ovarian cancer**	**Reference**
FGF18	Overexpression of FGF18 was identified as a predictive marker for poor clinical outcomes in patients with advanced stage, high-grade serous ovarian cancer.	[33]
HERC5	HERC5 was found to have increased expression levels in topotecan-resistant ovarian cancer cell lines.	[34]
Hsa-miR-202	MiR-202-5p was down-regulated in ovarian cancer and plays a role in suppressing cell proliferation, migration, and invasion in ovarian cancer.	[35]
RPS27A	The study [36] identified genes with survival-related alternative splicing events in ovarian cancer, and RPS27A was one of the hub genes in the gene interaction network.	[36]
**Key pathway factors**	**Pathway names**	**References****
R-HSA-168928	DDX58/IFIH1-mediated induction of interferon-alpha/beta.	[27]
R-HSA-936440	Negative regulators of DDX58/IFIH1 signaling.	[37]
R-HSA-72163	mRNA Splicing - Major Pathway.	[36,38]
R-HSA-1482788	Acyl chain remodeling of PC.	[39,40]
R-HSA-1482839	Acyl chain remodeling of PE.	[41]
R-HSA-2408557	Selenocysteine synthesis.	[42]

* **References for potential association with breast cancer or cancer in general**

** **References for potential association with ovarian cancer**

9. Identified key input factors and key pathway factors

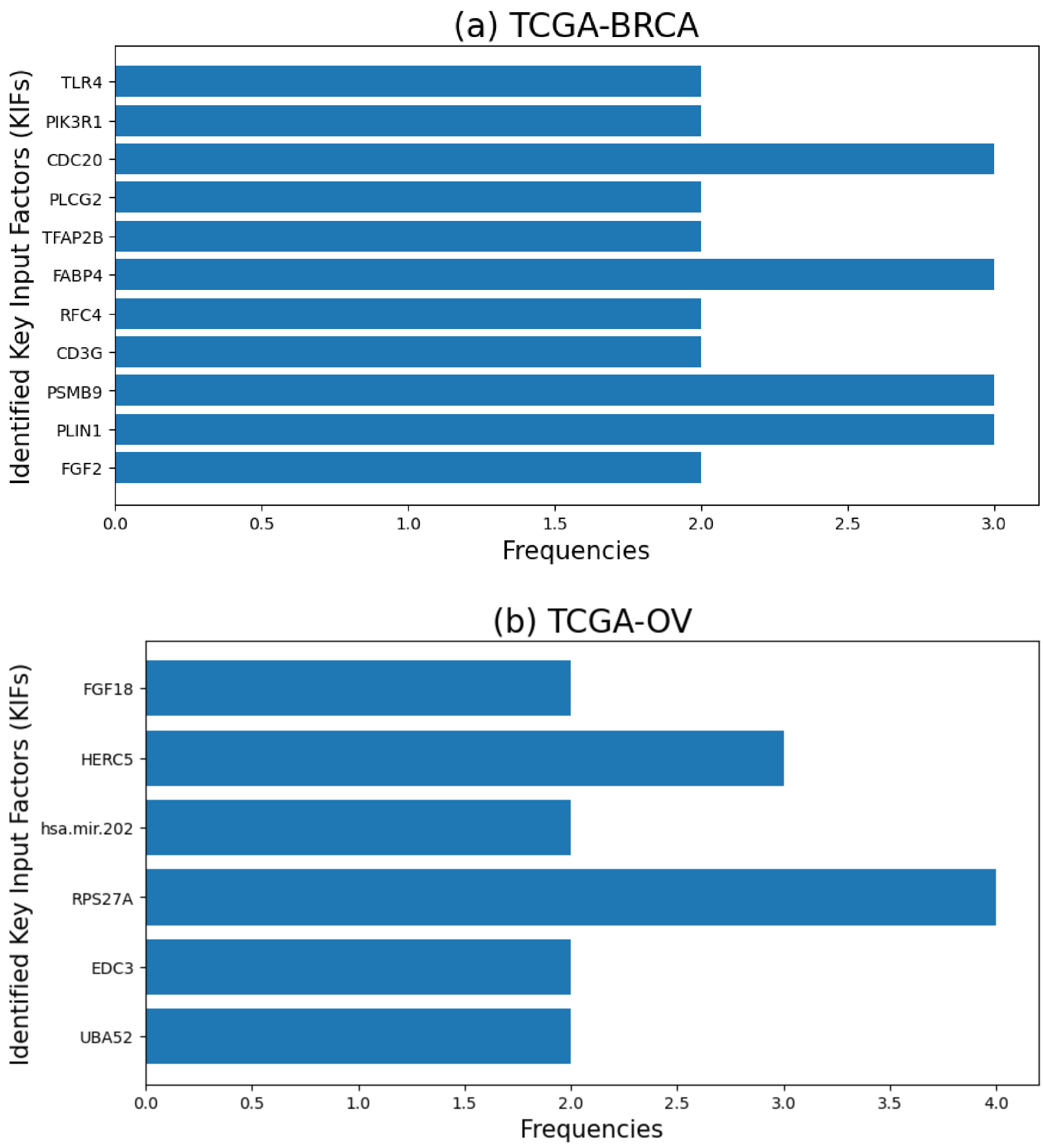

Supplementary Figure 6 – Identified Key input factors (KIFs). X-axis shows the names of input factors corresponding to (a) 11 genes for the TCGA-BRCA dataset, and (b) 5 genes and 1 miRNA for the TCGA-OV dataset. Y-axis shows the frequency for each gene/miRNA, that is, the number of latent features that included this gene/miRNA as one of their top 10 input factors that contributed most to their value differences between high- and low-risk groups.

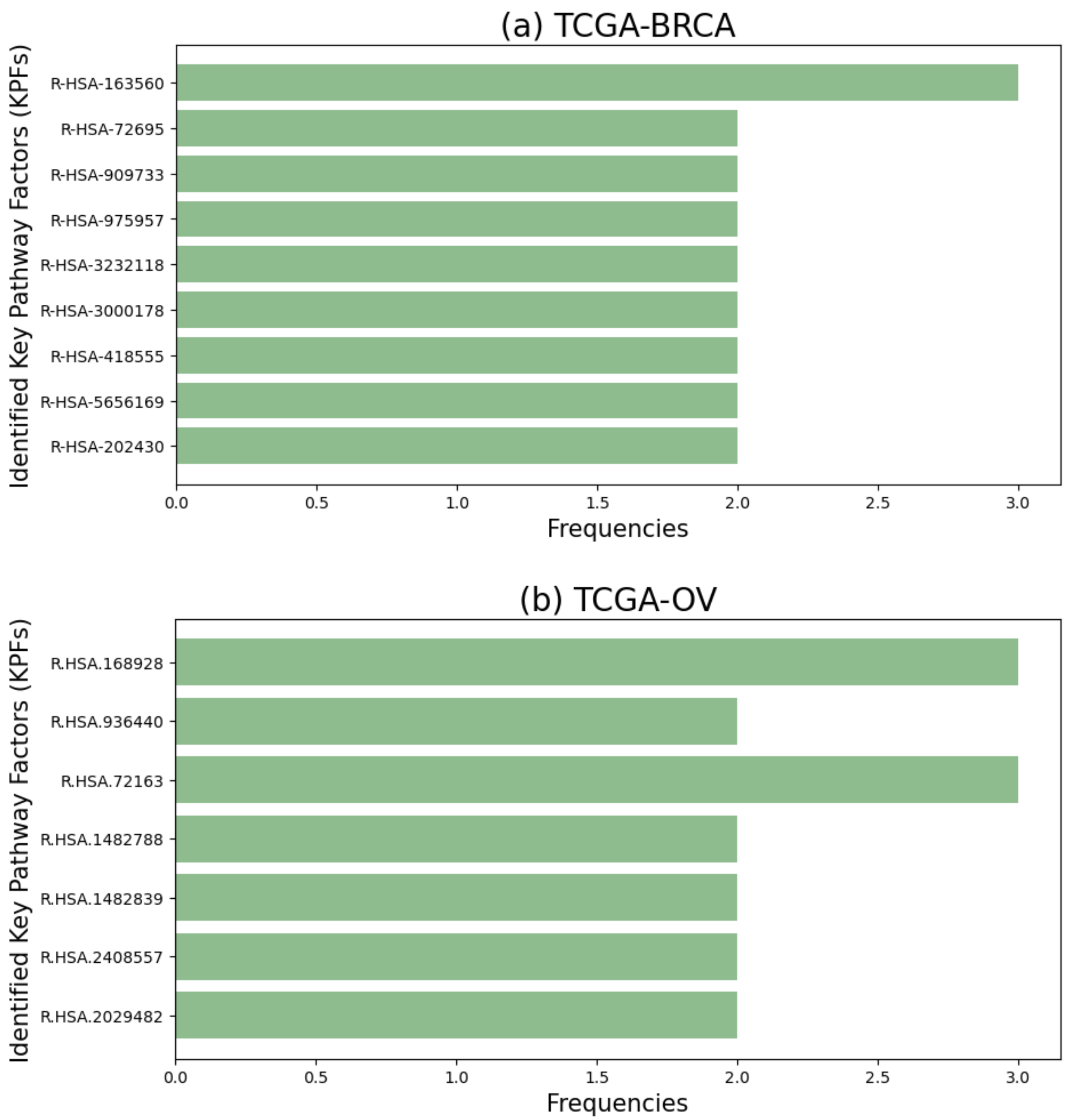

Supplementary Figure 7 - Identified key pathway factors (KPFs). X-axis shows the names of those pathways in (a) TCGA-BRCA dataset and (b) TCGA-OV dataset. Y-axis shows the frequency for each pathway, that is, the number of latent features that included this pathway as one of the top 10 pathways that contributed most to their value differences between high- and low-risk groups.

10. Univariate CoxPH analysis for identified key genes

Supplementary Table 6 – Summarized results from univariate CoxPH analyses for identified key genes

(a) TCGA-BRCA

Genes	Frequencies	HR	HR lower 95CI	HR upper 95CI	p-value	BH-adjusted p-value
TFAP2B	2	1.054	0.783	1.418	0.73	0.73
CDC20	3	0.778	0.576	1.051	0.1	0.22
TLR4	2	1.333	0.982	1.809	0.064	0.22
FGF2	2	1.181	0.874	1.597	0.277	0.311
PIK3R1	2	1.178	0.873	1.589	0.283	0.311
CD3G	2	0.763	0.565	1.03	0.076	0.22
RFC4	2	0.772	0.571	1.043	0.09	0.22
PLIN1	3	1.185	0.878	1.6	0.267	0.311
FABP4	3	1.23	0.909	1.663	0.178	0.280
PLCG2	2	1.258	0.93	1.702	0.135	0.248
PSMB9	3	0.716	0.531	0.967	0.029	0.22

(b) TCGA-OV

Genes	Frequencies	HR	HR lower 95CI	HR upper 95CI	p-value	BH-adjusted p-value
HERC5	3	0.892	0.685	1.162	0.397	0.662
RPS27A	4	0.993	0.762	1.293	0.957	0.957
FGF18	2	0.853	0.655	1.11	0.237	0.593
EDC3	2	1.225	0.94	1.596	0.134	0.593
UBA52	2	1.013	0.778	1.318	0.925	0.957

HR: Hazard ratio; HR lower 95CI - HR upper 95CI: the 95% confidence interval for HR. P-value obtained via likelihood ratio test.

11. Summarization of model performance

Supplementary Table 7 - Summarization of model performance during developmental stage and framework evaluation in different datasets

**Developmental stage**				
TCGA-BRCA				
	C-index (median/mean ± sd)			
	mRNA + miRNA +clinical	mRNA + clinical	miRNA + clinical	clinical only
AUTOSurv	**0.749 (0.75 ± 0.005)**	0.731 (0.729 ± 0.012)	0.738 (0.739 ± 0.01)	NA
AUTOSurv concat	0.737 (0.738 ± 0.012)	NA	NA	NA
AUTOSurv No KL	0.737 (0.735 ± 0.01)	0.734 (0.732 ± 0.013)	0.739 (0.738 ± 0.01)	NA
Surv-OmiVAE	0.74 (0.742 ± 0.009)	**0.736 (0.739 ± 0.008)**	**0.756 (0.754 ± 0.012)**	NA
Modified-SALMON	0.735 (0.732 ± 0.014)	0.734 (0.733 ± 0.013)	0.712 (0.712 ± 0.004)	NA
CoxPASNet	NA	0.653 (0.663 ± 0.036)	NA	NA
Cox-Enet	0.691 (0.691 ± 0.004)	0.686 (0.689 ± 0.015)	0.661 (0.641 ± 0.042)	NA
RSF	0.552 (0.554 ± 0.017)	0.563 (0.564 ± 0.019)	0.661 (0.663 ± 0.014)	NA
XGB-AFT	0.596 (0.593 ± 0.031)	0.614 (0.606 ± 0.04)	0.651 (0.645 ± 0.027)	NA
XGB-CoxPH	0.586 (0.588 ± 0.013)	0.615 (0.614 ± 0.019)	0.679 (0.674 ± 0.017)	NA
LFSurv	NA	NA	NA	0.714 (0.713 ± 0.008)
TCGA-OV				
	C-index (median/mean ± sd)			
	mRNA + miRNA +clinical	mRNA + clinical	miRNA + clinical	clinical only
AUTOSurv	**0.629 (0.628 ± 0.011)**	0.619 (0.62 ± 0.011)	**0.613 (0.61 ± 0.014)**	NA
AUTOSurv concat	0.611 (0.61 ± 0.019)	NA	NA	NA
AUTOSurv No KL	0.604 (0.606 ± 0.015)	0.613 (0.611 ± 0.015)	0.581 (0.581 ± 0.015)	NA
Surv-OmiVAE	0.601 (0.602 ± 0.022)	**0.629 (0.627 ± 0.012)**	0.595 (0.601 ± 0.014)	NA
Modified-SALMON	0.579 (0.581 ± 0.031)	0.613 (0.61 ± 0.014)	0.59 (0.584 ± 0.016)	NA
CoxPASNet	NA	0.599 (0.599 ± 0.004)	NA	NA
Cox-Enet	0.563 (0.555 ± 0.017)	0.574 (0.576 ± 0.008)	0.495 (0.522 ± 0.038)	NA
RSF	0.563 (0.563 ± 0.011)	0.578 (0.58 ± 0.014)	0.475 (0.474 ± 0.01)	NA
XGB-AFT	0.544 (0.548 ± 0.018)	0.582 (0.581 ± 0.013)	0.532 (0.527 ± 0.016)	NA
XGB-CoxPH	0.584 (0.586 ± 0.007)	0.602 (0.601 ± 0.012)	0.512 (0.511 ± 0.024)	NA
LFSurv	NA	NA	NA	0.623 (0.617 ± 0.015)

**Framework evaluation**				
ICGC-OVAU				
	C-index (median/mean ± sd)			
	mRNA + miRNA +clinical	mRNA + clinical	miRNA + clinical	clinical only
AUTOSurv	**0.735 (0.734 ± 0.033)**	**0.727 (0.72 ± 0.044)**	**0.711 (0.706 ± 0.051)**	NA
Modified-SALMON	0.691 (0.687 ± 0.045)	0.684 (0.681 ± 0.052)	0.695 (0.684 ± 0.064)	NA
Cox-Enet	0.582 (0.603 ± 0.058)	0.6 (0.608 ± 0.056)	0.582 (0.572 ± 0.048)	NA
RSF	0.62 (0.607 ± 0.071)	0.62 (0.605 ± 0.08)	0.548 (0.546 ± 0.044)	NA
XGB-AFT	0.521 (0.537 ± 0.075)	0.5 (0.532 ± 0.09)	0.5 (0.5 ± 0.084)	NA
XGB-CoxPH	0.648 (0.622 ± 0.1)	0.654 (0.604 ± 0.114)	0.561 (0.557 ± 0.095)	NA
LFSurv	NA	NA	NA	0.685 (0.681 ± 0.055)
Caldas-BC				
	C-index (median/mean ± sd)			
	mRNA + miRNA +clinical	mRNA + clinical	miRNA + clinical	clinical only
AUTOSurv	NA	0.78 (0.771 ± 0.066)	NA	NA
Modified-SALMON	NA	**0.839 (0.831 ± 0.071)**	NA	NA
Cox-Enet	NA	0.705 (0.701 ± 0.064)	NA	NA
RSF	NA	0.672 (0.676 ± 0.074)	NA	NA
XGB-AFT	NA	0.644 (0.639 ± 0.099)	NA	NA
XGB-CoxPH	NA	0.672 (0.675 ± 0.068)	NA	NA
LFSurv	NA	NA	NA	0.729 (0.725 ± 0.043)

- The highest median C-index in each case is highlighted in bold font.

Supplementary Table 8 - Summarization of model performance during external-cross-dataset validation

OV					
		C-index (median/mean ± sd)			
		mRNA + miRNA +clinical	mRNA + clinical	miRNA + clinical	clinical only
AUTOSurv	Internal validation	**0.697 (0.701 ± 0.034)**	0.673 (0.675 ± 0.024)	**0.678 (0.679 ± 0.026)**	NA
	External validation	**0.619 (0.624 ± 0.02)**	0.567 (0.567 ± 0.026)	**0.582 (0.58 ± 0.025)**	NA
Modified-SALMON	Internal validation	0.668 (0.677 ± 0.041)	**0.676 (0.681 ± 0.038)**	0.673 (0.672 ± 0.035)	NA
	External validation	0.595 (0.595 ± 0.03)	**0.595 (0.593 ± 0.025)**	0.549 (0.552 ± 0.012)	NA
Cox-Enet	Internal validation	0.626 (0.623 ± 0.04)	0.626 (0.624 ± 0.041)	0.623 (0.618 ± 0.036)	NA
	External validation	0.546 (0.551 ± 0.012)	0.55 (0.554 ± 0.014)	0.538 (0.537 ± 0.001)	NA
RSF	Internal validation	0.589 (0.598 ± 0.049)	0.588 (0.597 ± 0.042)	0.584 (0.578 ± 0.03)	NA
	External validation	0.577 (0.569 ± 0.015)	0.57 (0.565 ± 0.02)	0.555 (0.56 ± 0.012)	NA
XGB-AFT	Internal validation	0.555 (0.546 ± 0.038)	0.574 (0.584 ± 0.043)	0.577 (0.577 ± 0.061)	NA
	External validation	0.52 (0.521 ± 0.029)	0.499 (0.511 ± 0.033)	0.56 (0.557 ± 0.018)	NA
XGB-CoxPH	Internal validation	0.59 (0.598 ± 0.044)	0.583 (0.596 ± 0.044)	0.576 (0.571 ± 0.047)	NA
	External validation	0.551 (0.554 ± 0.014)	0.551 (0.545 ± 0.021)	0.566 (0.565 ± 0.008)	NA
LFSurv	Internal validation	NA	NA	NA	0.662 (0.65 ± 0.041)
	External validation	NA	NA	NA	0.54 (0.534 ± 0.026)
BRCA					
		C-index (median/mean ± sd)			
		mRNA + miRNA +clinical	mRNA + clinical	miRNA + clinical	clinical only
AUTOSurv	Internal validation	NA	**0.799 (0.794 ± 0.032)**	NA	NA
	External validation	NA	**0.709 (0.705 ± 0.024)**	NA	NA
Modified-SALMON	Internal validation	NA	0.795 (0.797 ± 0.032)	NA	NA
	External validation	NA	0.65 (0.651 ± 0.029)	NA	NA

Cox-Enet	Internal validation	NA	0.752 (0.747 ± 0.047)	NA	NA
	External validation	NA	0.58 (0.574 ± 0.015)	NA	NA
RSF	Internal validation	NA	0.739 (0.741 ± 0.042)	NA	NA
	External validation	NA	0.48 (0.48 ± 0.025)	NA	NA
XGB-AFT	Internal validation	NA	0.733 (0.7 ± 0.076)	NA	NA
	External validation	NA	0.504 (0.516 ± 0.085)	NA	NA
XGB-CoxPH	Internal validation	NA	0.753 (0.741 ± 0.048)	NA	NA
	External validation	NA	0.551 (0.553 ± 0.056)	NA	NA
LFSurv	Internal validation	NA	NA	NA	0.729 (0.736 ± 0.035)
	External validation	NA	NA	NA	0.672 (0.67 ± 0.006)

- The highest median C-index in each case is highlighted in bold font.
- OV: Models trained on TCGA-OV dataset and tested on ICGC-OVAU dataset; BRCA: Models trained on TCGA-BRCA dataset and tested on Caldas-BC dataset.
- Internal validation: Best internal validation model performance on the internal validation set during hyperparameter tuning; External validation: Model performance on the non-TCGA external validation datasets after trained on the TCGA datasets using the best hyperparameter sets.

References:

1. Naderi, A.*, et al.* A gene-expression signature to predict survival in breast cancer across independent data sets. *Oncogene* **26**, 1507-1516 (2007).
2. Akiba, T., Sano, S., Yanase, T., Ohta, T. & Koyama, M. Optuna: A next-generation hyperparameter optimization framework. in *Proceedings of the 25th ACM SIGKDD international conference on knowledge discovery & data mining* 2623-2631 (2019).
3. Mamoor, S. *CD3G is differentially expressed in the lymph nodes of patients with metastatic breast cancer*, (2021).
4. Karra, H.*, et al.* Cdc20 and securin overexpression predict short-term breast cancer survival. *British journal of cancer* **110**, 2905-2913 (2014).
5. Cheng, S., Castillo, V. & Sliva, D. CDC20 associated with cancer metastasis and novel mushroom-derived CDC20 inhibitors with antimetastatic activity. *International journal of oncology* **54**, 2250-2256 (2019).
6. Zeng, J., Sauter, E.R. & Li, B. FABP4: a new player in obesity-associated breast cancer. *Trends in molecular medicine* **26**, 437-440 (2020).
7. Suh, J., Kim, D.H., Lee, Y.H., Jang, J.H. & Surh, Y.J. Fibroblast growth factor‐2, derived from cancer‐associated fibroblasts, stimulates growth and progression of human breast cancer cells via FGFR1 signaling. *Molecular Carcinogenesis* **59**, 1028-1040 (2020).
8. Yan, L.-X.*, et al.* PIK3R1 targeting by miR-21 suppresses tumor cell migration and invasion by reducing PI3K/AKT signaling and reversing EMT, and predicts clinical outcome of breast cancer. *International journal of oncology* **48**, 471-484 (2016).
9. Rudolph, A.*, et al.* A comprehensive evaluation of interaction between genetic variants and use of menopausal hormone therapy on mammographic density. *Breast cancer research* **17**, 1-12 (2015).
10. Walker, L.C.*, et al.* Use of expression data and the CGEMS genome-wide breast cancer association study to identify genes that may modify risk in BRCA1/2 mutation carriers. *Breast cancer research and treatment* **112**, 229-236 (2008).
11. Zhou, C.*, et al.* Prognostic significance of PLIN1 expression in human breast cancer. *Oncotarget* **7**, 54488 (2016).
12. Rouette, A.*, et al.* Expression of immunoproteasome genes is regulated by cell-intrinsic and–extrinsic factors in human cancers. *Scientific reports* **6**, 34019 (2016).
13. Li, Y.*, et al.* Multifaceted regulation and functions of replication factor C family in human cancers. *American Journal of Cancer Research* **8**, 1343 (2018).
14. Fatima, A., Tariq, F., Malik, M.F.A., Qasim, M. & Haq, F. Copy number profiling of MammaPrint™ genes reveals association with the prognosis of breast cancer patients. *Journal of Breast Cancer* **20**, 246-253 (2017).
15. Fu, X.*, et al.* TFAP2B overexpression contributes to tumor growth and progression of thyroid cancer through the COX-2 signaling pathway. *Cell Death & Disease* **10**, 397 (2019).
16. Raap, M.*, et al.* Lobular carcinoma in situ and invasive lobular breast cancer are characterized by enhanced expression of transcription factor AP-2β. *Laboratory Investigation* **98**, 117-129 (2018).
17. Khademalhosseini, M. & Arababadi, M.K. Toll-like receptor 4 and breast cancer: an updated systematic review. *Breast Cancer* **26**, 265-271 (2019).
18. Holcik, M. Could the eIF2α-independent translation be the achilles heel of cancer? *Frontiers in oncology* **5**, 264 (2015).
19. Guo, L.*, et al.* Phosphorylated eIF2α predicts disease-free survival in triple-negative breast cancer patients. *Scientific reports* **7**, 44674 (2017).
20. Guaita‐Esteruelas, S.*, et al.* Adipose‐Derived Fatty Acid‐Binding Proteins Plasma Concentrations Are Increased in Breast Cancer Patients. *The Oncologist* **22**, 1309-1315 (2017).

21. Ray, G. & Husain, S.A. Role of lipids, lipoproteins and vitamins in women with breast cancer. *Clinical biochemistry* **34**, 71-76 (2001).
22. Chen, J., Moore, A. & Ringshausen, I. ZAP-70 shapes the immune microenvironment in B cell malignancies. *Frontiers in Oncology* **10**, 595832 (2020).
23. Yu, S., Sun, L., Jiao, Y. & Lee, L.T.O. The role of G protein-coupled receptor kinases in cancer. *International journal of biological sciences* **14**, 189 (2018).
24. Provance, O.K. & Lewis-Wambi, J. Deciphering the role of interferon alpha signaling and microenvironment crosstalk in inflammatory breast cancer. *Breast Cancer Research* **21**, 1-10 (2019).
25. Borden, E.C. Interferons α and β in cancer: therapeutic opportunities from new insights. *Nature Reviews Drug Discovery* **18**, 219-234 (2019).
26. Martin-Hijano, L. & Sainz Jr, B. The interactions between cancer stem cells and the innate interferon signaling pathway. *Frontiers in Immunology* **11**, 526 (2020).
27. Musella, M., Galassi, C., Manduca, N. & Sistigu, A. The yin and yang of type I IFNs in cancer promotion and immune activation. *Biology* **10**, 856 (2021).
28. Pawlicka, K., Kalathiya, U. & Alfaro, J. Nonsense-mediated mRNA decay: pathologies and the potential for novel therapeutics. *Cancers* **12**, 765 (2020).
29. Perrin-Vidoz, L., Sinilnikova, O.M., Stoppa-Lyonnet, D., Lenoir, G.M. & Mazoyer, S. The nonsense-mediated mRNA decay pathway triggers degradation of most BRCA1 mRNAs bearing premature termination codons. *Human molecular genetics* **11**, 2805-2814 (2002).
30. Tamayo-Angorrilla, M., de Andrés, J.L., Jiménez, G. & Marchal, J.A. The biomimetic extracellular matrix: A therapeutic tool for breast cancer research. *Translational Research* **247**, 117-136 (2022).
31. Qin, Y.*, et al.* SUMOylation wrestles with the occurrence and development of breast cancer. *Frontiers in Oncology* **11**, 659661 (2021).
32. Makridakis, N.M. & Reichardt, J.K. Translesion DNA polymerases and cancer. *Frontiers in genetics* **3**, 174 (2012).
33. Wei, W.*, et al.* FGF18 as a prognostic and therapeutic biomarker in ovarian cancer. *The Journal of clinical investigation* **123**, 4435-4448 (2013).
34. Swierczewska, M.*, et al.* New and old genes associated with primary and established responses to paclitaxel treatment in ovarian cancer cell lines. *Molecules* **23**, 891 (2018).
35. Yu, H. & Pan, S. MiR-202-5p suppressed cell proliferation, migration and invasion in ovarian cancer via regulating HOXB2. *Eur Rev Med Pharmacol Sci* **24**(2020).
36. Ouyang, Y.*, et al.* Alternative splicing acts as an independent prognosticator in ovarian carcinoma. *Scientific Reports* **11**, 10413 (2021).
37. Wolf, D.*, et al.* High RIG-I expression in ovarian cancer associates with an immune-escape signature and poor clinical outcome. *International journal of cancer* **146**, 2007-2018 (2020).
38. Yao, S.*, et al.* Alternative Splicing: A New Therapeutic Target for Ovarian Cancer. *Technology in Cancer Research & Treatment* **21**, 15330338211067911 (2022).
39. Iorio, E.*, et al.* Activation of phosphatidylcholine cycle enzymes in human epithelial ovarian cancer cells. *Cancer research* **70**, 2126-2135 (2010).
40. Zeleznik, O.A.*, et al.* Circulating lysophosphatidylcholines, phosphatidylcholines, ceramides, and sphingomyelins and ovarian cancer risk: a 23-year prospective study. *JNCI: Journal of the National Cancer Institute* **112**, 628-636 (2020).
41. Wang, Y.*, et al.* Serum lipid profiling analysis and potential marker discovery for ovarian cancer based on liquid chromatography–Mass spectrometry. *Journal of Pharmaceutical and Biomedical Analysis* **199**, 114048 (2021).
42. Farinella, F.*, et al.* Machine Learning analysis of high-grade serous ovarian cancer proteomic dataset reveals novel candidate biomarkers. *Scientific Reports* **12**, 3041 (2022).
